# Native GaN/GaO_x_ Heterostructure Platform for Wafer-Scale Integration of High-Performance Complementary Transistors

**DOI:** 10.34133/research.1058

**Published:** 2025-12-22

**Authors:** Jinhua Liang, Chi Liu, Yuning Wei, Shun Feng, Yun Sun, Yutaka Ohno, Hui-Ming Cheng, Dong-Ming Sun

**Affiliations:** ^1^ Shenyang National Laboratory for Materials Science, Institute of Metal Research, Chinese Academy of Sciences, Shenyang 110016, China.; ^2^ School of Materials Science and Engineering, University of Science and Technology of China, Shenyang 110016, China.; ^3^ Department of Quantum Engineering, Nagoya University, Nagoya 464-8603, Japan.; ^4^ Faculty of Materials Science and Engineering/Institute of Technology for Carbon Neutrality, Shenzhen Institute of Advanced Technology, Chinese Academy of Sciences, Shenzhen 518000, China.

## Abstract

The drive for complementary transistors with ultra-steep subthreshold swing (SS) and minimal power consumption has intensified interest in integrating low-dimensional semiconductors with wide-bandgap materials, as silicon scaling approaches its physical limits in the post-Moore era. A central bottleneck, however, lies in dielectric/semiconductor interface engineering: existing strategies based on van der Waals transfer or nonnative dielectric deposition compromise interface quality, wafer-scale uniformity, and process compatibility, preventing practical large-scale integration. Here, we demonstrate a previously unexplored in situ oxidation strategy that directly forms a high-κ gallium oxide (GaO_x_) dielectric on heavily doped n-type gallium nitride (GaN) substrates, enabling high-performance, energy-efficient complementary inverters based on n-type molybdenum disulfide (MoS_2_) and p-type carbon nanotube transistors. Leveraging the excellent dielectric properties of the GaN/GaO_x_ heterostructure, MoS_2_ field-effect transistors achieve an interface trap density of 1.18 × 10^11^ cm^−2^ eV^−1^, an SS at the thermionic limit of 60 mV dec^−1^, an on/off ratio above 10^8^ at 0.87 V, and a voltage gain of 134.5 at 2 V for inverters. These results establish GaN/GaO_x_ as a native, scalable, and complementary metal-oxide-semiconductor-compatible integration platform, offering a transformative route toward wafer-scale, low-power electronics in the post-Moore era.

## Introduction

In the post-Moore era, as the scaling of traditional silicon-based transistors approaches its fundamental physical limits, severe short-channel effects lead to sharply increased off-state current and power dissipation, which severely constrain the further enhancement of integrated circuit performance [1–5]. The development of novel complementary transistors with an ultra-steep subthreshold swing (approaching the theoretical limit of 60 mV dec^−1^) and ultralow static power consumption has consequently emerged as a critical challenge. The wide-bandgap semiconductor gallium nitride (GaN), known for its high breakdown field strength, high electron mobility, and excellent thermal stability, provides a highly promising platform for heterogeneous integration with emerging low-dimensional materials such as molybdenum disulfide (MoS_2_) and carbon nanotubes (CNTs), whose atomic-scale thickness enables superior gate controllability and suppressed off-state leakage current. This material system presents a compelling route to simultaneously achieve superior gate controllability, high carrier mobility, and intrinsically low power consumption, opening new possibilities for next-generation integrated electronics [6–10]. However, a major bottleneck in this integration scheme lies in the poor control over the interface quality between low-dimensional materials and dielectric layers [11–13]. Conventional atomic layer deposition (ALD) of high-κ dielectrics (Al_2_O_3_ and HfO_2_) often suffers from nonuniform nucleation on 2-dimensional (2D) material surfaces due to the lack of surface dangling bonds, leading to inferior interface quality that degrades subthreshold characteristics and operational stability at scaled equivalent oxide thickness (EOT) [14,15]. To address this interfacial challenge, several integration strategies have been explored, including transferred van der Waals dielectrics (such as HfO_x_ and h-BN) [16–18], molecular buffer interlayers (such as Sb_2_O_3_ and 3,4,9,10-perylene-tetracarboxylic dianhydride [PTCDA]) [19–21], and epitaxially grown crystalline dielectrics (such as CaF_2_) [22] via molecular beam epitaxy. While these approaches have yielded quantifiable improvements in interface quality, their practical application is often constrained by inherent technical limitations, such as difficulties in achieving thickness uniformity in van der Waals dielectrics and the incompatibility of high-temperature epitaxial growth processes with standard CMOS technology, which hinders the wafer-scale uniformity and process compatibility required for reliable large-scale integration in post-Moore electronics.

Here, we propose an innovative strategy of in situ growth: through controlled thermal oxidation of heavily doped n-type GaN substrates, a high-quality, high-κ gallium oxide (GaO_x_) dielectric layer is directly generated on the GaN surface. This method leverages the innate properties of GaN to construct a high-performance GaN/GaO_x_ heterostructure platform with excellent dielectric properties. MoS_2_ field-effect transistors (FETs) fabricated on this GaO_x_ dielectric show an interface state density as low as 1.18 × 10^11^ cm^−2^ eV^−1^ and a small subthreshold swing of 60 mV dec^−1^. The GaN/GaO_x_ heterostructure platform is also compatible with high-performance p-type CNT FETs. Fabricated complementary inverters integrating n-type MoS_2_ and p-type CNT transistors achieve a maximum voltage gain of 134.5 at a supply voltage of 2 V, which demonstrates the potential of this technology for future low-power integrated circuits.

## Results

### Device design and characterization

**Fig. 1.**
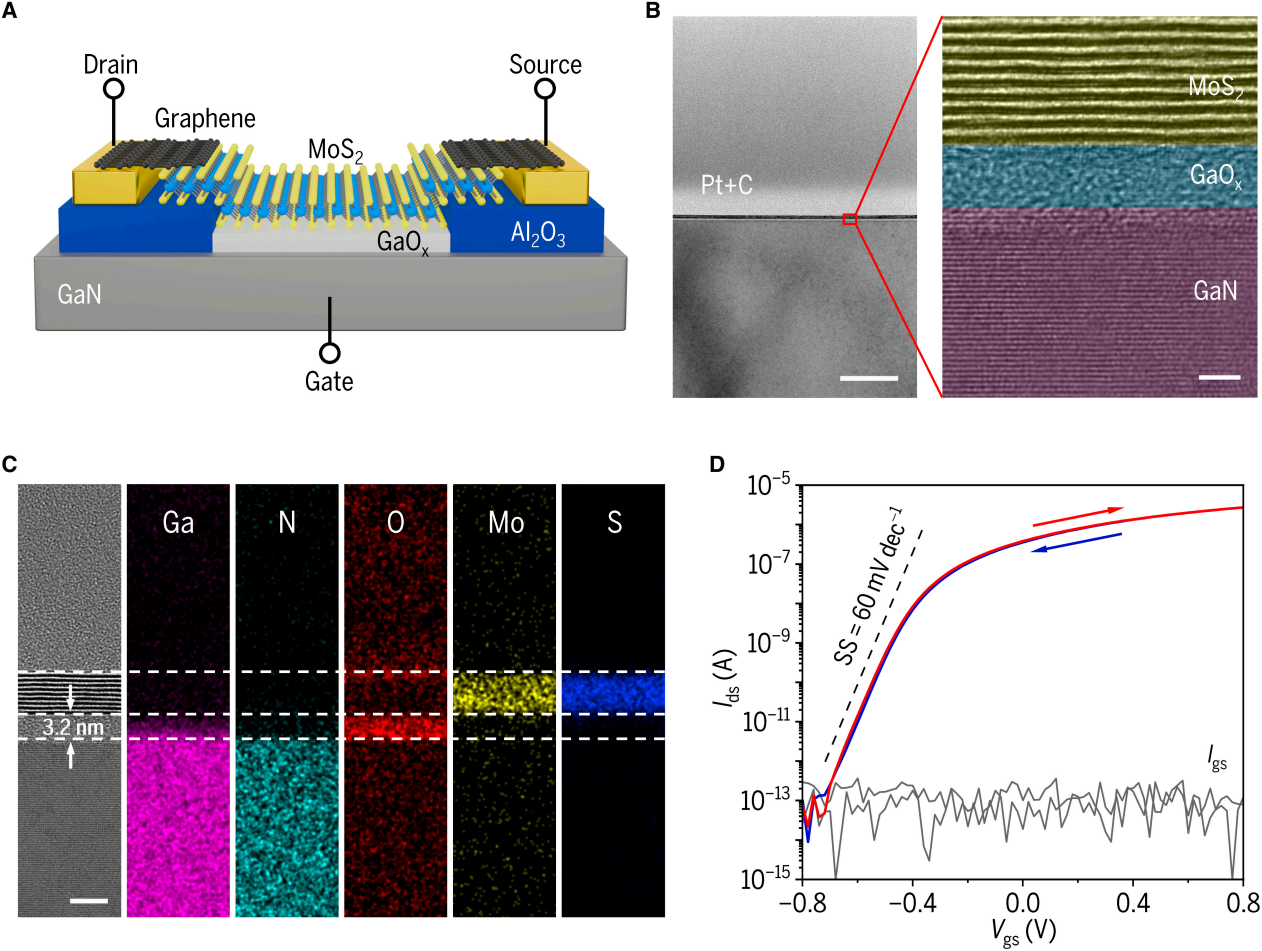
Design and fabrication of device. (A) Schematic diagram of the MoS_2_ transistor. (B) Cross-sectional TEM image of the MoS_2_/GaO_x_/GaN heterostructure showing a high-quality interface between MoS_2_ and GaO_x_ (left scale bar: 100 nm, right scale bar: 2 nm). (C) TEM image with corresponding elemental mappings of Ga, N, O, Mo, and S, respectively (scale bar: 5 nm). (D) Double-sweep transfer characteristic curves of the MoS_2_ transistor with a constant *V*_ds_ of 1 V, featuring a channel length of 20 μm and a width of 5 μm.

Figure 1A shows the schematic diagram of an MoS_2_ transistor with a back-gate stack of MoS_2_/GaO_x_/GaN, where the GaO_x_ dielectric is prepared by thermal oxidation of the GaN substrate. A 30-nm-thick Al_2_O_3_ is used as the isolation layer. The Ti/Al/Ti/Au stack serves as the gate electrode and enables an ohmic contact with the GaN substrate (Fig. S1), while graphene is used as source and drain electrodes [23]. Detailed fabrication processes are shown in Figs. S2 and S3. The cross-sectional transmission electron microscopy (TEM) image of the gate stack clearly reveals a uniform 3.2-nm-thick GaO_x_ dielectric with atomically flat surfaces (Fig. 1B), while the elemental mapping (Fig. 1C) and the Raman spectra (Fig. S4) collectively confirm the gate stack composition. This high-quality GaN/GaO_x_ heterostructure platform enables exceptional MoS_2_ transistor performance, including a large on/off ratio over 10^8^, a small gate leakage current below 100 fA, and a near-ideal subthreshold swing (SS) of 60 mV dec^−1^ in double-sweep transfer characteristic curves (Fig. 1D).

### High-κ GaO_x_ dielectric layer

**Fig. 2.**
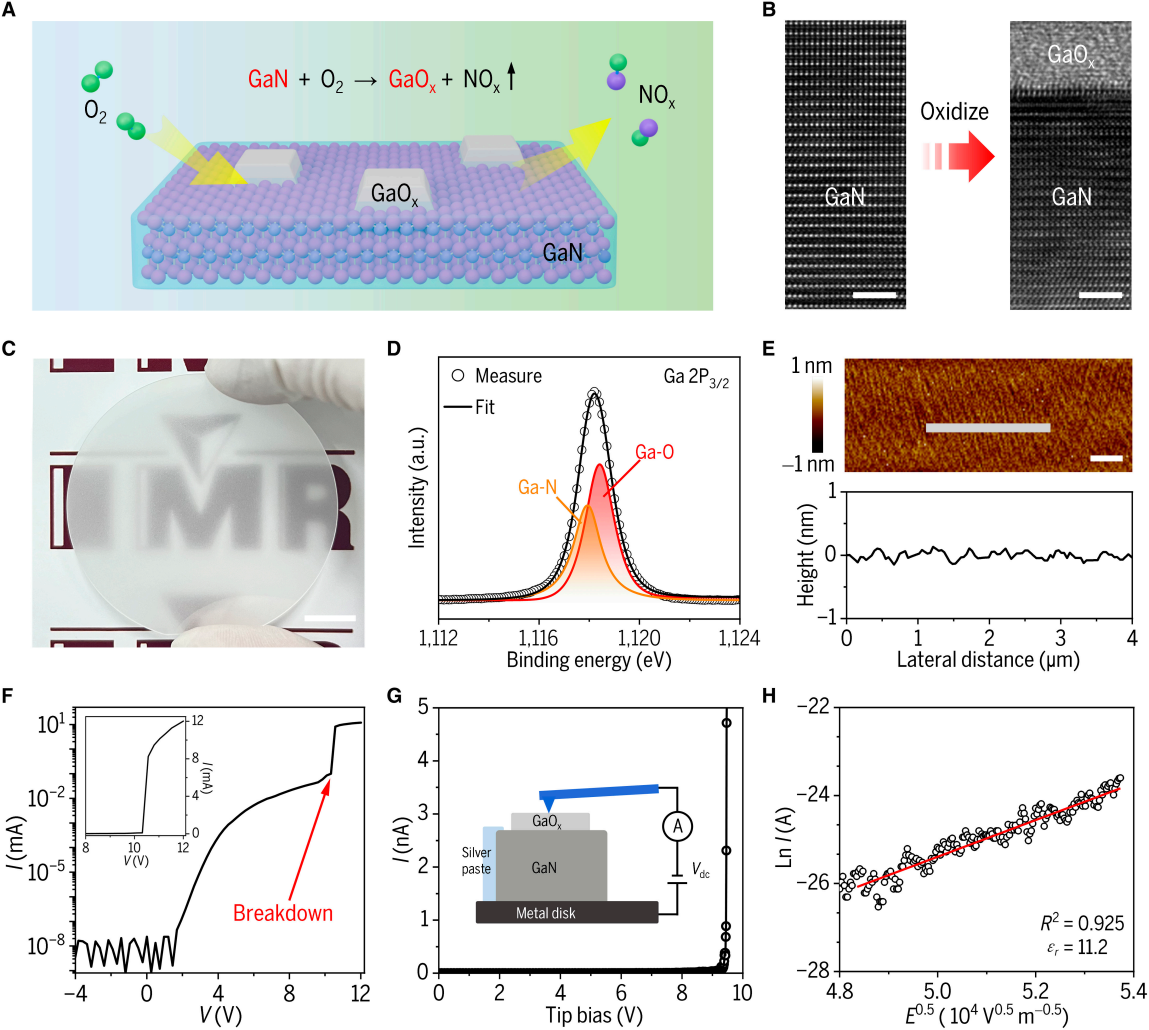
Preparation and property of GaO_x_ dielectric. (A) Schematic diagram of GaO_x_ formation mechanism via thermal oxidation of the GaN (0001) substrate. (B) Cross-sectional TEM image of the in situ grown GaO_x_ dielectric on GaN substrate (scale bar: 2 nm). (C) Optical image of a 2-inch wafer with uniform GaO_x_ film (scale bar: 1 cm). (D) High-resolution XPS spectra of Ga 2P_3/2_ core level for the GaO_x_ dielectric (prepared at 300 °C for 60 min), fitted by Ga-N (low-intensity) and Ga-O (high-intensity) bonding states. (E) AFM image of the GaO_x_ dielectric (scale bar: 1 μm) and corresponding height profile. (F) Current leakage and breakdown characteristics of the GaO_x_ film. (G) Current–voltage (*I–V*) characteristics of the GaO_x_ layer. Inset: Schematic diagram of the setup for the peak force tunneling AFM (PF-TUNA) measurement. (H) Schottky emission model fitting of *I–V* data for dielectric constant extraction.

The GaO_x_ dielectric layer is fabricated through thermal oxidation of GaN in an oxygen-rich environment. During this process, oxygen atoms progressively replace nitrogen atoms in the surface layers, forming GaO_x_ with tiny-grained oxide while releasing NO_x_ gas (Fig. 2A and B) [24–26]. Importantly, this thermal oxidation process enables wafer-scale production of GaO_x_ dielectrics (Fig. 2C). X-ray photoelectron spectroscopy (XPS) is used to analyze the chemical composition of the GaO_x_ film. The experimental Ga 2P_3/2_ core-level spectrum is well-fitted by both Ga-N and Ga-O bonding configurations (Fig. 2D), confirming the formation of Ga-O components in the dielectric while residual Ga-N signatures remain detectable due to the ultrathin nature of the dielectric [27,28]. Consistent conclusions are obtained from the Ga 3d core-level spectrum (Fig. S5). Complementary measurements of secondary ion mass spectrometry (SIMS) further verify that the dielectric layer consists of pure Ga-O bonding, with negligible Ga-N content (Fig. S6). Atomic force microscopy (AFM) characterization demonstrates that the GaO_x_ dielectric exhibits exceptional uniformity and flatness at the micrometer scale, showing no discernible surface protrusions or holes (Fig. 2E). The high-quality interface enables the GaO_x_ dielectric to achieve complete insulation below 2 V (Fig. 2F and Fig. S7).

The dielectric constant of the GaO_x_ dielectric is characterized by an AFM-based PeakForce TUNA technology, which has been widely used to measure the dielectric properties of ultrathin materials [29,30]. A schematic diagram of the measurement is illustrated in Fig. 2G. Current–voltage (*I–V*) characteristics are measured by applying a sweeping bias voltage to the sample. The curve demonstrates that the GaO_x_ film is completely insulated below 7.7 V, and the breakdown point where the current rises above the machine noise level is about 9.3 V. Using the Schottky emission model to fit and analyze the results, we calculate a dielectric constant of 11.2 for the GaO_x_ layer, corresponding to an EOT of 1.1 nm (Fig. 2H). In addition, repetitive measurements across multiple locations on the same sample and different samples are conducted, and similar results confirm the excellent uniformity of the GaO_x_ dielectric (Fig. S8). We also conduct Au/GaO_x_/GaN MOSCAP measurements with double sweep at different frequencies and scan rates, and the observation of negligible hysteresis under all test conditions confirms the exceptional stability and reliability of the GaO_x_ dielectric layer (Fig. S9).

The effects of oxidation temperature (200 to 500 °C) and time were examined to clarify their role in the dielectric formation mechanism [31,32]. XPS analysis shows progressive shifts of the Ga 2P_3/2_ and Ga 3d peaks toward higher binding energies by 0.14 and 0.16 eV (Fig. S10) with concurrent bandgap reduction from 4.9 to 4.8 eV (Fig. S11), confirming temperature-dependent oxidation. The insulation performance of the GaO_x_ dielectric is highly dependent on the thermal activation process. Insufficient treatment (low temperature/short time) results in incomplete oxidation, whereas excessive conditions (high temperature/prolonged time) generate numerous grain boundaries. These grain boundaries serve as leakage pathways for carriers, and both extremes ultimately degrade the insulation properties, as evidenced in Figs. S12 and S13.

### High-performance MoS_2_ transistor

**Fig. 3.**
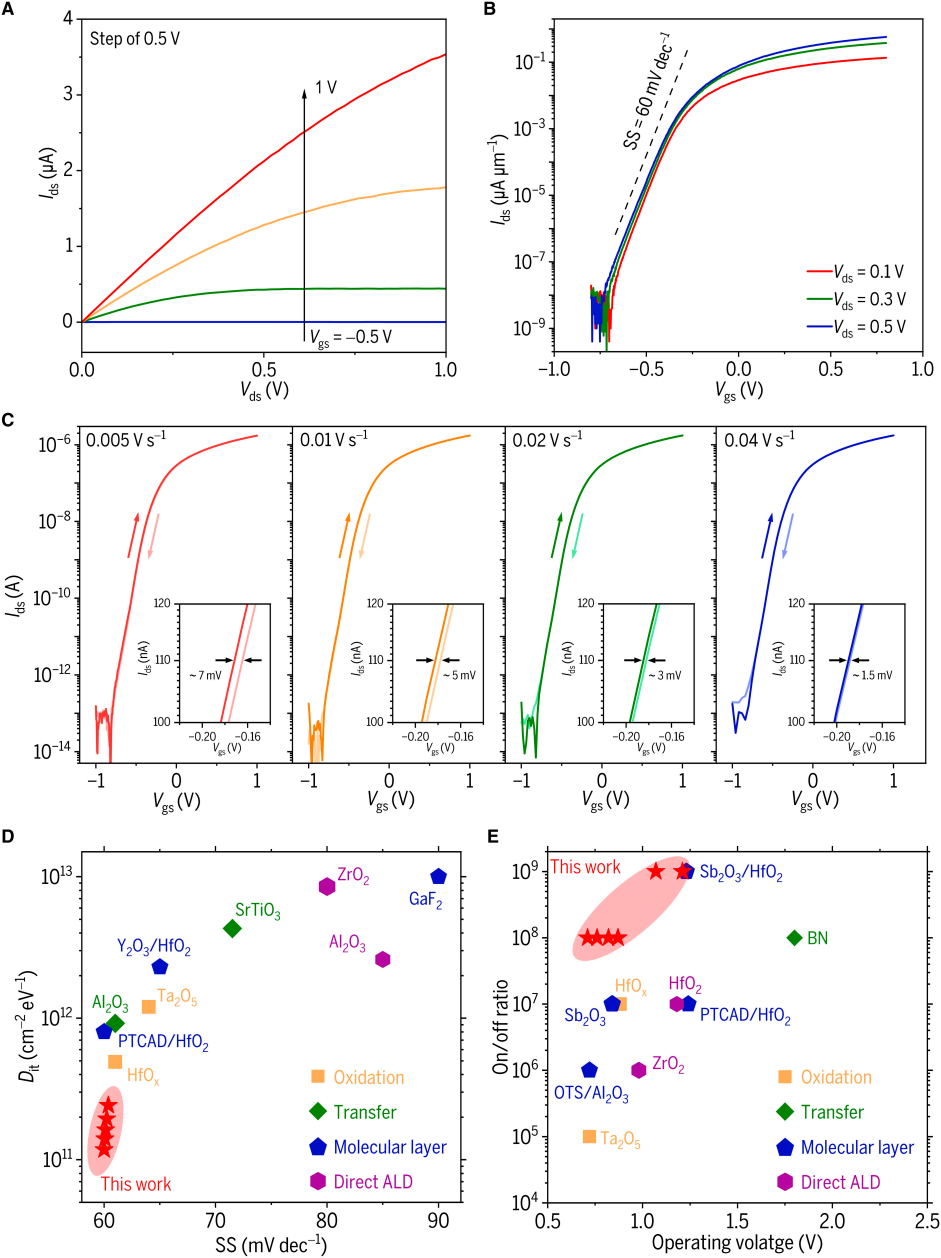
Excellent performance of MoS_2_ FET fabricated on a GaN/GaO_x_ heterostructure platform. (A) Output characteristic curves of MoS_2_ transistor with *V*_gs_ increasing from −0.5 to 1 V at a step size of 0.5 V. (B) Transfer characteristic curves of MoS_2_ transistor at different drain voltages, featuring a channel length of 7 μm and a width of 5 μm. (C) Double-sweep transfer characteristic curves of the MoS_2_ transistor at various sweep rates with a constant *V*_ds_ of 0.5 V. (D) Subthreshold swing (SS) and interface state density (*D*_it_) of our device in comparison with transistors fabricated by various approaches [18,21,22,34–39]*.* (E) Operating voltage and on/off ratio of our device in comparison with MoS_2_ transistors fabricated by various approaches [16,19–21,37,40–43].

Owing to the high-quality interface and small EOT of the GaO_x_ dielectric, the MoS_2_ transistors fabricated on a GaN/GaO_x_ heterostructure platform exhibit excellent electrical characteristics, including high on/off current ratios at different drain voltages, especially a high on/off current ratio over 10^8^ under an operating voltage of 0.87 V when *V*_ds_ = 0.5 V (Fig. 3A and B). Similar excellent performance is also demonstrated in other devices (Fig. S14).

Additionally, negligible hysteresis (~7 mV) is observed in the reverse gate voltage scan, which is slightly influenced by the sweeping speed (Fig. 3C), signifying the high-quality MoS_2_/GaO_x_ interface with a low interface state density (*D*_it_). The *D*_it_ can be evaluated using the equation [17,33]:

$$D_{\mathrm{it}}=\frac{C_{G}}{q}\left(\frac{q\mathrm{SS}}{k_{B}T\text{In}10}-1\right)$$ (1)

where *C*_G_ is the capacitance per unit area of the gate, SS is the subthreshold swing, *q* is the elementary charge of the electron, $k_{B}$ is the Boltzmann constant, and *T* is the temperature in kelvin. A *D*_it_ as low as 1.18 × 10^11^ cm^−2^ eV^−1^ is evaluated. Furthermore, both the multiple switching cycle tests and the bias stress stability tests confirm the superior interface quality of the GaO_x_ dielectric layer, as evidenced by a negligible threshold voltage shift (Δ*V*_th_ < 10 mV) in the MoS_2_ transistors (Figs. S15 and S16).

To benchmark the performance of our device, the key parameters such as *D*_it_, SS, and on/off ratio are compared with the state-of-the-art MoS_2_ transistors employing various dielectric integration strategies (Fig. 3D and E). These strategies encompass several established methods, including direct ALD of Al_2_O_3_ or ZrO_2_, molecular-layer-assisted ALD with Sb_2_O_3_ or PTCDA, material oxidation, and the transfer of van der Waals dielectrics like BN or SrTiO_3_. In contrast, the GaN/GaO_x_ heterostructure platform adopted in this work exhibits an ultralow *D*_it_ (1.18 × 10^11^ cm^−2^ eV^−1^), enabling superior device characteristics: a near-ideal subthreshold swing of 60 mV dec^−1^ and a high on/off ratio of 10^8^ at ultralow operating voltages. These results underscore the exceptional interface quality of the MoS_2_/GaO_x_ heterostructure, highlighting its marked potential for future low-power 2D device applications.

### MoS_2_ and CNT transistor arrays

**Fig. 4.**
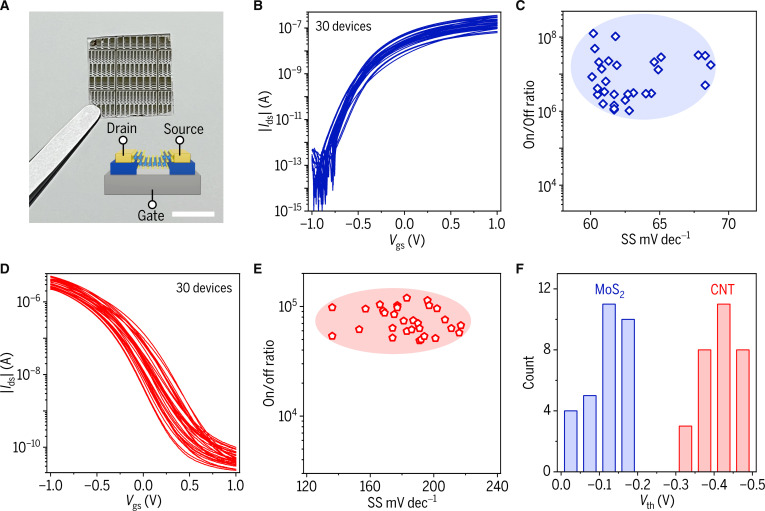
MoS_2_ and CNT transistor arrays. (A) Optical image of CVD MoS_2_ transistor arrays fabricated on a GaN/GaO_x_ heterostructure platform (scale bar: 0.5 cm). Inset: Schematic diagram of the CVD MoS_2_ transistor. (B and C) Transfer characteristic curves of 30 MoS_2_ transistors with the same channel length (B), and their statistics of SS and on/off ratio (C). (D and E) Transfer characteristic curves of 30 CNT transistors with the same channel length (D), and their statistics of SS and on/off ratio (E). (F) Statistical distribution of *V*_th_ for 30 CVD MoS_2_ FETs and 30 CNT FETs.

To investigate the potential applications of the GaN/GaO_x_ heterostructure platform, we have fabricated the FET arrays based on large-area monolayer CVD MoS_2_ and wafer-scale GaN/GaO_x_ heterostructure platform (Fig. 4A), following similar fabrication procedures with the transistors of exfoliated MoS_2_ (further details are provided in Fig. S2). The CVD MoS_2_ FETs exhibit excellent consistency in transfer characteristic curves at different channel lengths (Fig. S17) under a *V*_ds_ of −0.5 V (Fig. 4B and C).

While the MoS_2_ transistor arrays exhibit excellent n-type characteristics (on/off ratio over 10^7^, subthreshold swing ~ 60 mV dec^−1^), it is equally imperative to realize high-performance p-type devices for the development of complementary logic architectures. One-dimensional (1D) CNTs, with their inherent hole-transport superiority and compatibility with low-temperature processing*,* serve as an ideal counterpart to match with n-type MoS_2_ (Fig. S18). Additionally, CNT films are compatible with uniform and scalable fabrication processes, enabling the deposition of high-quality, highly homogeneous thin films over large areas. This capability provides a reliable material foundation for the mass production of high-performance electronic devices (Fig. S19). Utilizing this GaN/GaO_x_ heterostructure platform, we successfully fabricated CNT transistor arrays and conducted comprehensive electrical characterization of 30 devices with identical channel lengths (*L* = 30 μm) at *V*_ds_ = −1 V (Fig. 4D and E). The CNT transistors show improved gate control (Fig. S20) with GaO_x_ dielectric. Statistical analysis of threshold voltages (*V*_th_) for both MoS_2_ and CNT transistors exhibits narrow Gaussian distributions (Fig. 4F), confirming the excellent uniformity and reproducible fabrication of the GaN/GaO_x_ heterostructure platform with different semiconductor materials.

### Complementary inverter

As the fundamental building block of CMOS circuits, the voltage transfer characteristics (voltage gain) of inverters are intrinsically governed by the electrostatic control capability of the dielectric layer.

**Fig. 5.**
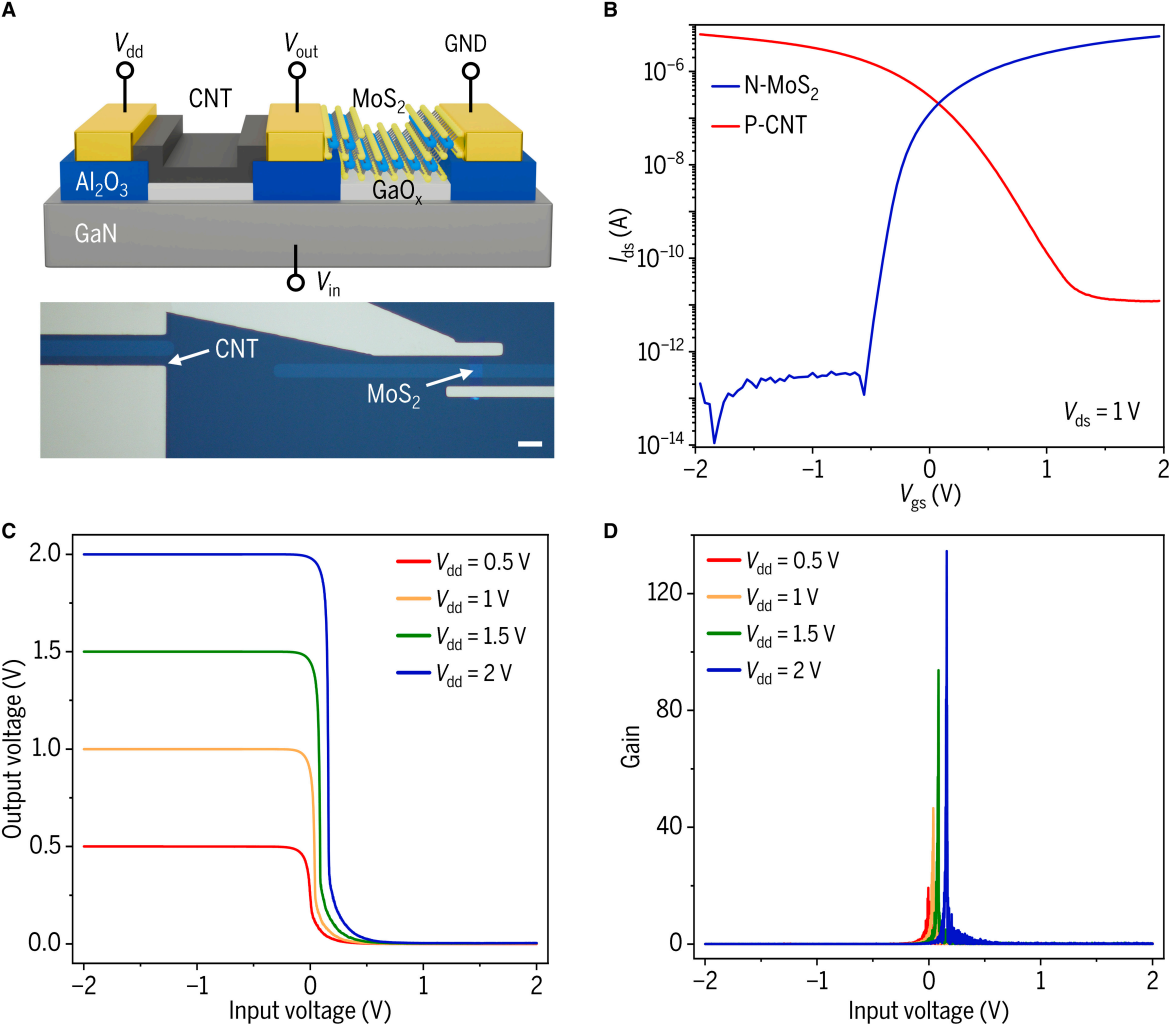
Complementary inverter fabricated with n-type MoS_2_ FET and p-type CNT FET. (A) Schematic diagram and optical image of the inverter fabricated on a GaN/GaO_x_ heterostructure platform (scale bar: 10 μm). (B) Transfer characteristic curves of MoS_2_ and CNT transistors. (C) The voltage transfer characteristics of the inverter at various supply voltages. (D) Corresponding voltage gains.

In this work, we successfully employed high-κ GaO_x_ as the gate dielectric to achieve co-integration of n-type MoS_2_ and p-type CNT transistors on a GaN/GaO_x_ heterostructure platform. This strategy demonstrates excellent electrical matching performance and enables the fabrication of complementary inverters (Fig. 5A and B). The voltage transfer characteristic (*V*_out_−*V*_in_) and voltage gain (d*V*_out_/d*V*_in_) are measured by sweeping the input voltage (*V*_in_) from −2 to 2 V under different supply voltages (*V*_dd_), as depicted in Fig. 5C and D, respectively. Utilizing the superior gate control of GaO_x_ dielectric, the inverter exhibits sharp switching near *V*_in_ = 0 V across all *V*_dd_ values with *V*_out_ infinitely close to *V*_dd_ or 0 V. Notably, a maximum voltage gain of 134.5 is achieved at *V*_dd_ = 2 V. The rapid switching response provides compelling evidence for superior gate control enabled by our GaN/GaO_x_ heterostructure platform.

Furthermore, dynamic switching cycle tests were conducted on the fabricated inverters. The results indicated that the output voltage amplitude and waveform maintained excellent consistency over consecutive cycles, demonstrating remarkable operational stability (Fig. S21). Further benchmarking against other state-of-the-art complementary inverters reported in the literature revealed that our devices exhibit competitive performance metrics, providing strong evidence for the practical reliability of the proposed platform in integrated circuits (Fig. S22).

## Discussion

In conclusion, we have developed an excellent wafer-scale high-κ GaO_x_ dielectric through the thermal oxidation of GaN and leveraged it for the integration of 2D and 1D materials. MoS_2_ FETs fabricated on a GaN/GaO_x_ heterostructure exhibit excellent performance, including an ultralow *D*_it_ of 1.18 × 10^11^ cm^−2^ eV^−1^ and a high on/off current ratio of 10^8^ under a small operating voltage of 0.87 V. The heterostructure platform simultaneously supports the fabrication of both CVD-grown monolayer MoS₂ and CNT FET arrays, demonstrating excellent reproducibility across multiple transistors. Complementary inverters integrating n-type MoS_2_ and p-type CNT transistors achieve a record-high voltage gain of 134.5 at *V*_dd_ = 2 V. Our work presents a versatile and CMOS-compatible heterostructure integration platform that can unlock the potential of 2D/1D materials for advanced high-performance complementary electronics.

## Materials and Methods

### Fabrication of a GaN/GaO_x_ heterostructure

The heavily doped n-type GaN substrate (0001) (resistivity: <0.05 Ω cm) was cleaned sequentially in acetone and isopropanol for 5 min each to remove surface contaminants, followed by diluted HF cleaning for 30 s. The GaO_x_ dielectric was prepared by thermal oxidation in an oxygen atmosphere at 300 °C for 60 min.

### Fabrication of MoS_2_ FETs on a GaN/GaO_x_ heterostructure platform

After ultrasonic cleaning and acid cleaning, a photoresist layer (LOR3A) was spin-coated at 2,000 revolutions per minute (rpm) for 60 s on the GaN substrate and baked at 190 °C for 5 min. Then, another photoresist layer (S-1813) was spin-coated at 4,000 rpm for 60 s on the substrate and baked at 120 °C for 2 min. An undercut structure was fabricated by photolithography and developing processes. The ohmic contact between the metal electrodes and the GaN substrate was formed using a Ti/Al/Ti/Au (20/30/20/30 nm) stack deposited by electron-beam evaporation and lift-off, with subsequent annealing at 700 °C for 30 s in N_2_. Then, the Al_2_O_3_ layer (30 nm thick) was deposited on the substrate via ALD at 150 °C, using trimethylaluminum (TMA) and water as precursors. Metal contacts (Ti/Au: 5/50 nm) of drain and source electrodes were deposited on the Al_2_O_3_ layer. Then, Al_2_O_3_ layer was patterned by photolithography and etched with H_3_PO_4_ at 70 °C for 3 min, leaving windows on the GaN substrate for GaO_x_ dielectric formation. A few-layer MoS_2_ flake was mechanically exfoliated from a bulk crystal and transferred onto a polydimethylsiloxane stamp. It was then released onto the target GaO_x_ dielectric using an electronic van der Waals transfer station. With the same method, we transferred 2 graphene flakes onto the substrate to connect MoS_2_ and the Ti/Au and completed the fabrication of the FET.

### Fabrication of CVD MoS_2_ and CNT FET arrays on a GaN/GaO_x_ heterostructure platform

The fabrication of CVD MoS_2_ and CNT FET arrays followed initial steps similar to the exfoliated MoS_2_ transistors. After establishing ohmic contacts to the GaN substrate, an Al_2_O_3_ layer (30 nm thick) was deposited and patterned via photolithography and H_3_PO_4_ etching, followed by GaO_x_ dielectric formation in the exposed regions. For MoS_2_ FETs, a centimeter-scale continuous monolayer CVD MoS_2_ film was first transferred onto the prepatterned substrate. Then, the excess MoS_2_ film was removed by oxygen plasma treatment with an oxygen flow rate of 180 standard cubic centimeters per minute (sccm) for 2 min. Metal contacts (Ti/Au: 5/50 nm) of drain and source electrodes were deposited on the MoS_2_ film using photolithography and electron beam evaporation. For CNT FETs, the drain and source electrodes (Ti/Au: 5/50 nm) were first deposited on Al_2_O_3_. A semiconducting CNT film was then deposited from a toluene solution at 60 °C over 2 h, followed by sequential cleaning in toluene and isopropyl alcohol at 80 °C for 10 min each. Finally, CNT channels were patterned and etched using oxygen plasma treatment at 200 W with an oxygen flow rate of 180 sccm for 2 min.

### Fabrication of complementary inverter

The complementary inverter is implemented with an n-type and a p-type transistor configured in a complementary architecture. First, the p-type CNT FET was fabricated following the standard processes described above. Subsequently, a MoS_2_ flake was transferred onto the GaO_x_ dielectric adjacent to the CNT device, followed by deposition of source/drain electrodes (Ti/Au: 5/50 nm) to complete the n-type MoS_2_ FET. Finally, the p-CNT and n-MoS_2_ FETs were electrically interconnected to form a complementary inverter.

### Characterization

The fabricated devices were characterized using an optical microscope (Nikon ECLIPSE LV100ND), TEM (FEI Talos F200X), XPS (Thermo VG Scientific ESCA LAB250), AFM (Bruker Dimension Icon AFM), micro-Raman analyzer (Jobin Yvon HR800 with 532-nm laser excitation), SIMS (ION TOF-SIMS 5), and x-ray diffraction. The electrical performance of the transistors was measured using a semiconductor analyzer (Agilent B1500A equipped with a B1500A-A20 capacitance measurement unit) and probe station (Cascade Microtech, 150-PK-PROMOTION).

## Data Availability

The study’s data can be obtained from the corresponding authors upon reasonable request.

## References

[1] Fiori G, Bonaccorso F, lannaccone G, Palacios T, Neumaier D, Seabaugh A, Banejee SK, Colombo L. Electronics based on two-dimensional materials. Nat Nanotechnol. 2014;9(10):768–779. 25286272 10.1038/nnano.2014.207

[2] Akinwande D, Huyghebaert C, Wang CH, Serna MI, Goossens S, Li LJ, Wong HSP, Koppens FHL. Graphene and two-dimensional materials for silicon technology. Nature. 2019;573(7775):507–518. 31554977 10.1038/s41586-019-1573-9

[3] Liu C, Ma W, Chen ML, Ren WC, Sun DM. A vertical silicon-graphene-germanium transistor. Nat Commun. 2019;10:4873. 31653842 10.1038/s41467-019-12814-1 PMC6814790

[4] Zhuo FL, Wu J, Li BH, Li MY, Tan C, Luo ZZ, Sun HB, Xu Y, Yu ZH. Modifying the power and performance of 2-dimensional MoS_2_ field effect transistors. Research. 2023;6: Article 0057. 36939429 10.34133/research.0057 PMC10016345

[5] Ieong M, Doris B, Kedzierski J, Rim K, Yang M. Silicon device scaling to the sub-10-nm regime. Science. 2004;306(5704):2057–2060. 15604400 10.1126/science.1100731

[6] Wu F, Tian H, Shen Y, Hou Z, Ren J, Guo GY, Sun YB, Yang Y, Ren TL. Vertical MoS_2_ transistors with sub-1-nm gate lengths. Nature. 2022;603(7900):259–264. 35264756 10.1038/s41586-021-04323-3

[7] Franklin A. Nanomaterials in transistors: From high-performance to thin-film applications. Science. 2015;349(6249):aab2750. 26273059 10.1126/science.aab2750

[8] Zheng Z, Zhang L, Song W, Feng S, Xu H, Sun J, Yang S, Chen T, Wei J, Chen KJ. Gallium nitride-based complementary logic integrated circuits. Nat Electron. 2021;4(8):595–603.

[9] Liu LJ, Han J, Xu L, Zhou JS, Zhao CY, Ding SJ, Shi HW, Xiao MM, Ding L, Ma Z, et al. Aligned, high-density semiconducting carbon nanotube arrays for high-performance electronics. Science. 2020;368(6498):850–856. 32439787 10.1126/science.aba5980

[10] Peng J, Chen ZJ, Ding BF, Cheng HM. Recent advances for the synthesis and applications of 2-dimensional ternary layered materials. Research. 2023;6: Article 0040. 37040520 10.34133/research.0040 PMC10076031

[11] Lin YX, Lin CM, Chen HY, Vaziri S, Bao XY, Woon WY. Dielectric material technologies for 2-d semiconductor transistor scaling. IEEE Tran Elec Dev. 2023;70(4):1454–1473.

[12] Liu AH, Peng XY, Peng SG, Tian H. Dielectrics for 2-d electronics: From device to circuit applications. IEEE Tran Elec Dev. 2023;70(4):1474–1498.

[13] Yang SJ, Liu KL, Xu YS, Liu LX, Li HQ, Zhai TY. Gate dielectrics integration for 2d electronics: Challenges, advances, and outlook. Adv Mater. 2023;35(18):2207901. 10.1002/adma.202207901 36226584

[14] Wang JL, Li SL, Zou XM, Ho J, Liao L, Xiao XH, Jiang CZ, Hu WD, Wang JL, Li JC. Integration of high-κ oxide on MoS_2_ by using ozone pretreatment for high-performance MoS_2_ top-gated transistor with thickness-dependent carrier scattering investigation. Small. 2015;11(44):5932–5938. 26426344 10.1002/smll.201501260

[15] Kim HG, Lee HBR. Atomic layer deposition on 2d materials. Chem Mater. 2017;29(9):3809–3826.

[16] Vu QA, Fan SD, Lee SH, Joo MK, Yu WJ, Lee YH. Near-zero hysteresis and near-ideal subthreshold swing in h-BN encapsulated single-layer MoS_2_ field-effect transistors. 2D Mater. 2018;5(3): Article 031001.

[17] Britnell L, Gorbachev RV, Jalil R, Belle BD, Schedin F, Katsnelson MI, Eaves L, Morozov SV, Mayorov AS, Peres NMR, et al. Electron tunneling through ultrathin boron nitride crystalline barriers. Nano Lett. 2012;12(3):1707–1710. 22380756 10.1021/nl3002205

[18] Jing YM, Dai XF, Yang JQ, Zhang XB, Wang ZW, Liu XC, Li HM, Yuan YH, Zhou XF, Luo H, et al. Integration of ultrathin hafnium oxide with a clean van der waals interface for two-dimensional sandwich hetlerostructure electronics. Nano Lett. 2024;24(13):3937–3944. 38526847 10.1021/acs.nanolett.4c00117

[19] Xu YS, Liu T, Liu KL, Zhao YH, Lei L, Li PH, Nie AM, Liu LX, Yu J, Feng X, et al. Scalable integration of hybrid high-κ dielectric materials on two-dimensional semiconductors. Nat Mater. 2023;22(9):1078–1084. 37537352 10.1038/s41563-023-01626-w

[20] Li WS, Zhou J, Cai SH, Yu ZH, Zhang JL, Fang N, Li TT, Wu Y, Chen TS, Xie XY, et al. Uniform and ultrathin high-κ gate dielectrics for two-dimensional electronic devices. Nat Electron. 2019;2(12):563–571.

[21] Park JH, Fathipour S, Kwak I, Sardashti K, Ahles CF, Wolf SF, Edmonds M, Vishwanath S, Xing HG, Fullerton-Shirey SK, et al. Atomic layer deposition of Al_2_O_3_ on WSe_2_ functionalized by titanyl phthalocyanine. ACS Nano. 2016;10(7):6888–6896. 27305595 10.1021/acsnano.6b02648

[22] Illarionov YY, Banshchikov AG, Polyushkin DK, Wachter S, Knobloch T, Thesberg M, Mennel L, Paur M, Stöger-Pollach M, Steiger-Thirsfeld A. Ultrathin calcium fluoride insulators for two-dimensional field-effect transistors. Nat Electron. 2019;2(6):230–235.

[23] Greco G, Iucolano F, Roccaforte F. Ohmic contacts to gallium nitride materials. Appl Surf Sci. 2016;383(10):324–345.

[24] Janicki Ł, Korbutowicz R, Rudziński M, Michałowski P, Złotnik S, Grodzicki M, Gorantla S, Serafińczuk J, Hommel D, Kudrawiec R. Thermal oxidation of [0001] GaN in water vapor compared with dry and wet oxidation: Oxide properties and impact on GaN. Appl Surf Sci. 2022;598(10): Article 153872.

[25] Han YR, Wang YF, Xia DY, Fu SH, Gao C, Ma JG, Xu HY, Li BS, Shen AD, Liu YC. Rapid response solar blind deep uv photodetector with high detectivity based on graphene:N/βGa_2_O_3_:N/GaN p-i-n heterojunction fabricated by a reversed substitution growth method. Small Methods. 2023;7(7):2300041. 10.1002/smtd.202300041 37096880

[26] Li XX, Wu ZF, Fang Y, Huang SQ, Fang CZ, Wang YB, Zeng XY, Yang YG, Hao Y, Liu Y. Ga_2_O_3_ solar-blind deep-ultraviolet photodetectors with a suspended structure for high responsivity and high-speed applications. Research. 2024;7: Article 0546. 39664294 10.34133/research.0546 PMC11632154

[27] Su T, Xiao BH, Ai ZK, Bao LJ, Chen WC, Shen YH, Cheng Q, Ostrikov KK. High-rate growth of gallium oxide films by plasma-enhanced thermal oxidation for solar-blind photodetectors. Appl Surf Sci. 2023;624(7): Article 157162.

[28] Yamamoto T, Taoka N, Ohta A, Truyen NX, Yamada H, Takahashi T, Ikeda M, Makihara K, Shimizu M, Miyazaki S. Low-temperature formation of Ga-oxide/GaN interface with remote oxygen plasma and its interface properties. Jpn J Appl Phys. 2018;57(6S2):06JE01.

[29] Jahanmir J, West PE, Rhodin TN. Evidence of schottky emission in scanning tunneling microscopes operated in ambient air. Appl Phys Lett. 1988;52(6):2086–2088.

[30] Zavabeti A, Ou JZ, Carey BJ, Syed N, Orrell-Trigg R, Mayes ELH, Xu C, Kavehei O, O’Mullane AP, Kaner RB. A liquid metal reaction environment for the room-temperature synthesis of atomically thin metal oxides. Science. 2017;358(6361):332–335. 29051372 10.1126/science.aao4249

[31] Huang R, Liu T, Zhao YF, Zhu YF, Huang ZL, Li FS, Liu JP, Zhang LQ, Zhang SM, Sun AD, et al. Angular dependent xps study of surface band bending on Ga-polar n-GaN. Appl Surf Sci. 2018;440(5):637–642.

[32] Kumar SS, Rubio EJ, Noor-A-Alam M, Martinez G, Manandhar S, Shutthanandan V, Thevuthasan S, Ramana CV. Structure, morphology, and optical properties of amorphous and nanocrystalline gallium oxide thin films. J Phys Chem C. 2013;117(8):4194–4200.

[33] Yoshioka H, Senzaki J, Shimozato A, Tanaka Y, Okumura H. Effects of interface state density on 4H-SiC n-channel field-effect mobility. Appl Phys Lett. 2014;104(2): Article 083516.

[34] Huang JK, Wan Y, Shi JJ, Zhang J, Wang ZH, Wang WX, Yang N, Liu Y, Lin CH, Guan XW, et al. High-κ perovskite membranes as insulators for two-dimensional transistors. Nature. 2022;605(5):262–267. 35546188 10.1038/s41586-022-04588-2

[35] Zeng D, Zhang Z, Xue Z, Zhang M, Chu PK, Mei Y, Tian Z, Di Z. Single-crystalline metal-oxide dielectrics for top-gate 2d transistors. Nature. 2024;632(8):788–794. 39112708 10.1038/s41586-024-07786-2 PMC11338823

[36] Zou XM, Wang JL, Chiu CH, Wu Y, Xiao XH, Jiang CZ, Wu WW, Mai LQ, Chen TS, Li JC, et al. Interface engineering for high-performance top-gated MoS_2_ field-effect transistors. Adv Mater. 2014;26(36):6255–6261. 25070646 10.1002/adma.201402008

[37] Chamlagain B, Cui QS, Paudel S, Cheng MMC, Chen PY, Zhou ZX. Thermally oxidized 2d TaS_2_ as a high-κ gate dielectric for MoS_2_ field-effect transistors. 2D Dent Mater. 2017;4(3): Article 031002.

[38] Wang X, Zhang TB, Yang W, Zhu H, Chen L, Sun QQ, Zhang DW. Improved integration of ultra-thin high-κ dielectrics in few FET by remote forming gas plasma pretreatment. Appl Phys Lett. 2017;110(5): Article 053110.

[39] Yan H, Wang Y, Li Y, Phuyal D, Liu LX, Hl G, Guo YZ, Lee T, Kim M, Heong HY, et al. A clean van der Waals interface between the high-κ dielectric zirconium oxide and two-dimensional molybdenum disulfide. Nat Electron. 2025;8(10):906–912. 41158978 10.1038/s41928-025-01468-1 PMC12554778

[40] Luo PF, Liu C, Lin J, Duan XP, Zhang WJ, Ma C, Lv YW, Zou XM, Liu Y, Schwierz F, et al. Molybdenum disulfide transistors with enlarged van der waals gaps at their dielectric interface via oxygen accumulation. Nat Electron. 2022;5(12):849–858.

[41] Zhu YB, Li YJ, Arefe G, Burke RA, Tan C, Hao YF, Liu XC, Liu X, Yoo WJ, Dubey M, et al. Monolayer molybdenum disulfide transistors with single-atom-thick gates. Nano Lett. 2018;18(6):3807–3813. 29768000 10.1021/acs.nanolett.8b01091

[42] Desai SB, Madhvapathy SR, Sachid AB, Llinas JP, Wang QX, Ahn GH, Pitner G, Kim MJ, Bokor J, Hu CM, et al. MoS_2_ transistors with 1-nanometer gate lengths. Science. 2016;354(6308):99–102. 27846499 10.1126/science.aah4698

[43] Liu KL, Jin B, Han W, Chen X, Gong PL, Huang L, Zhao YH, Li L, Yang SJ, Hu XZ, et al. A wafer-scale van der waals dielectric made from an inorganic molecular crystal film. Nat Electron. 2021;4(11):906–913.

