# Supplementary material for: Native GaN/GaOx Heterostructure Platform for Wafer-Scale Integration of High-Performance Complementary Transistors
Source: Research (Wash D C). 2025 Dec 22;8:1058. doi: 10.34133/research.1058 (PMC12719557; doi:10.34133/research.1058)
Supplement: Supplementary 1 — Figs. S1 to S22 [file research.1058.f1.docx]

Supplementary Materials for

**Native GaN/GaO_x_ heterostructure platform for wafer-scale integration of high-performance complementary transistors**

Jinhua Liang *et al.*

Corresponding author: Chi Liu, chiliu@imr.ac.cn; Dongming Sun, dmsun@imr.ac.cn

**This PDF file includes:**

Figs. S1 to S22

**
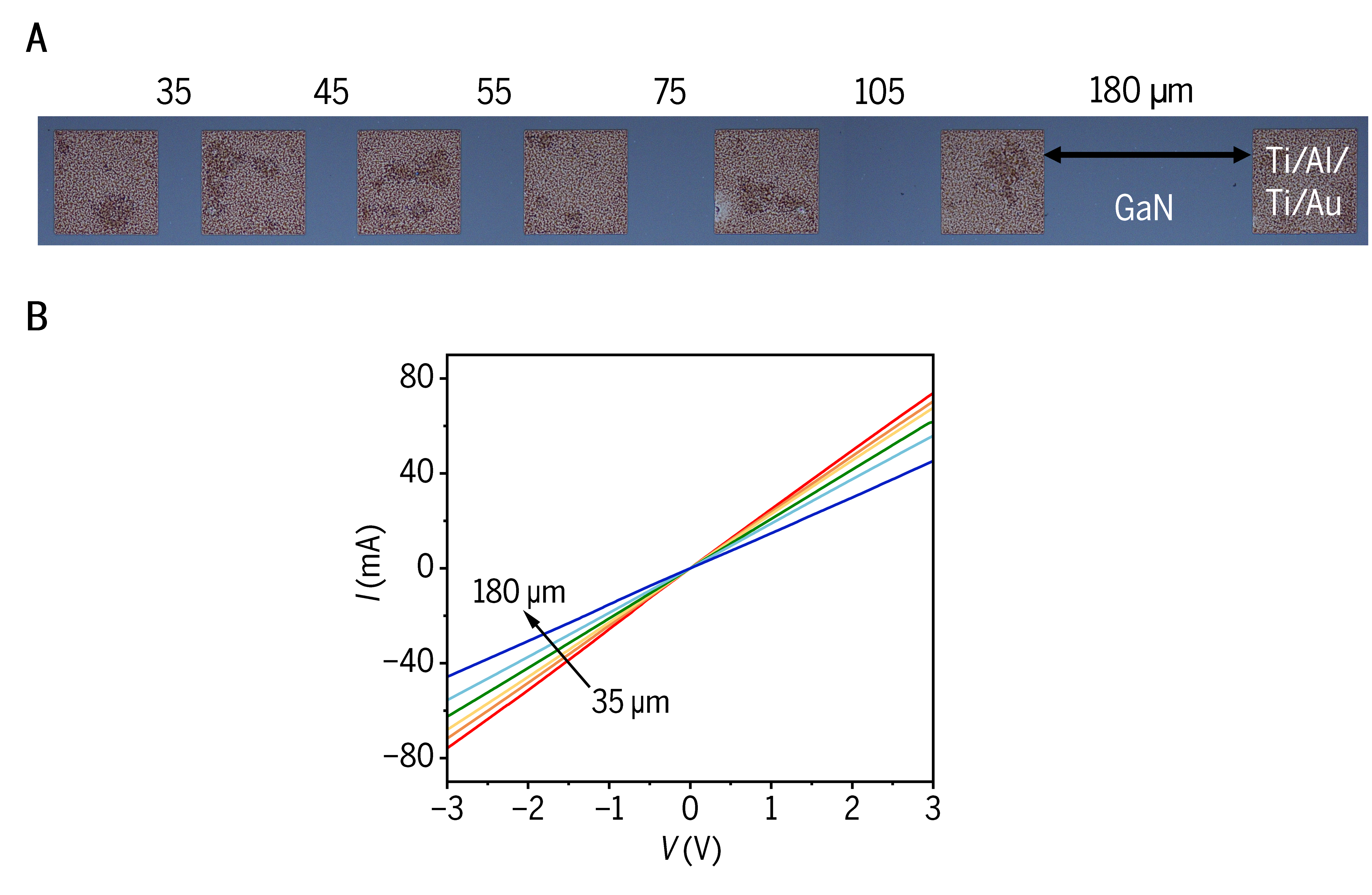
**

**Fig. S1.** **Ohmic contact between GaN substrate and electrode.** (A) Optical micrograph of electrodes on GaN substrate in different distances of 35, 45, 55, 75, 105 and 180 µm. (B) *I-V* characteristics between adjacent electrodes.


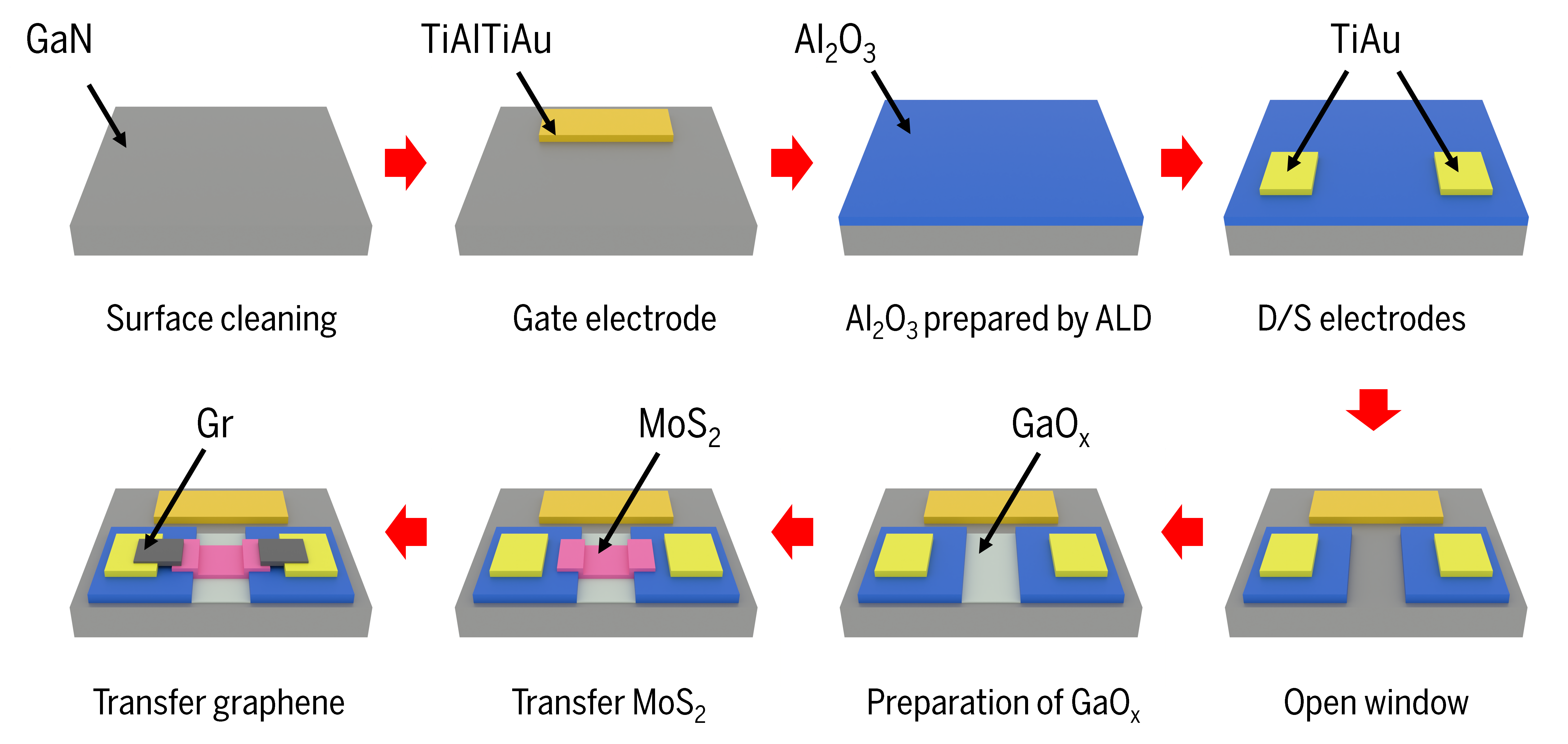


**Fig. S2.** **Device fabrication process.**

After ultrasonic cleaning and acid cleaning, metal contacts (Ti/Al/Ti/Au: 20/30/20/30 nm) were formed on GaN substrate by electron-beam evaporation and lift-off processes. Then the substrate was annealed and Ohmic contact was obtained between the electrodes and GaN. A 30-nm-thick Al_2_O_3_ layer was deposited on the substrate by atomic layer deposition (ALD). Metal contacts (Ti/Au: 5/50 nm) of drain and source electrodes were deposited. The Al_2_O_3_ layer was patterned by photolithography and etched with H_3_PO_4_, leaving windows on the GaN substrate followed by the preparation of GaO_x_ dielectric. Few-layer MoS_2_ flake was transferred onto the target GaO_x_ dielectric. Two graphene flakes were transferred onto the substrate to connect MoS_2_ and the Ti/Au and completed the fabrication of the FET.

**
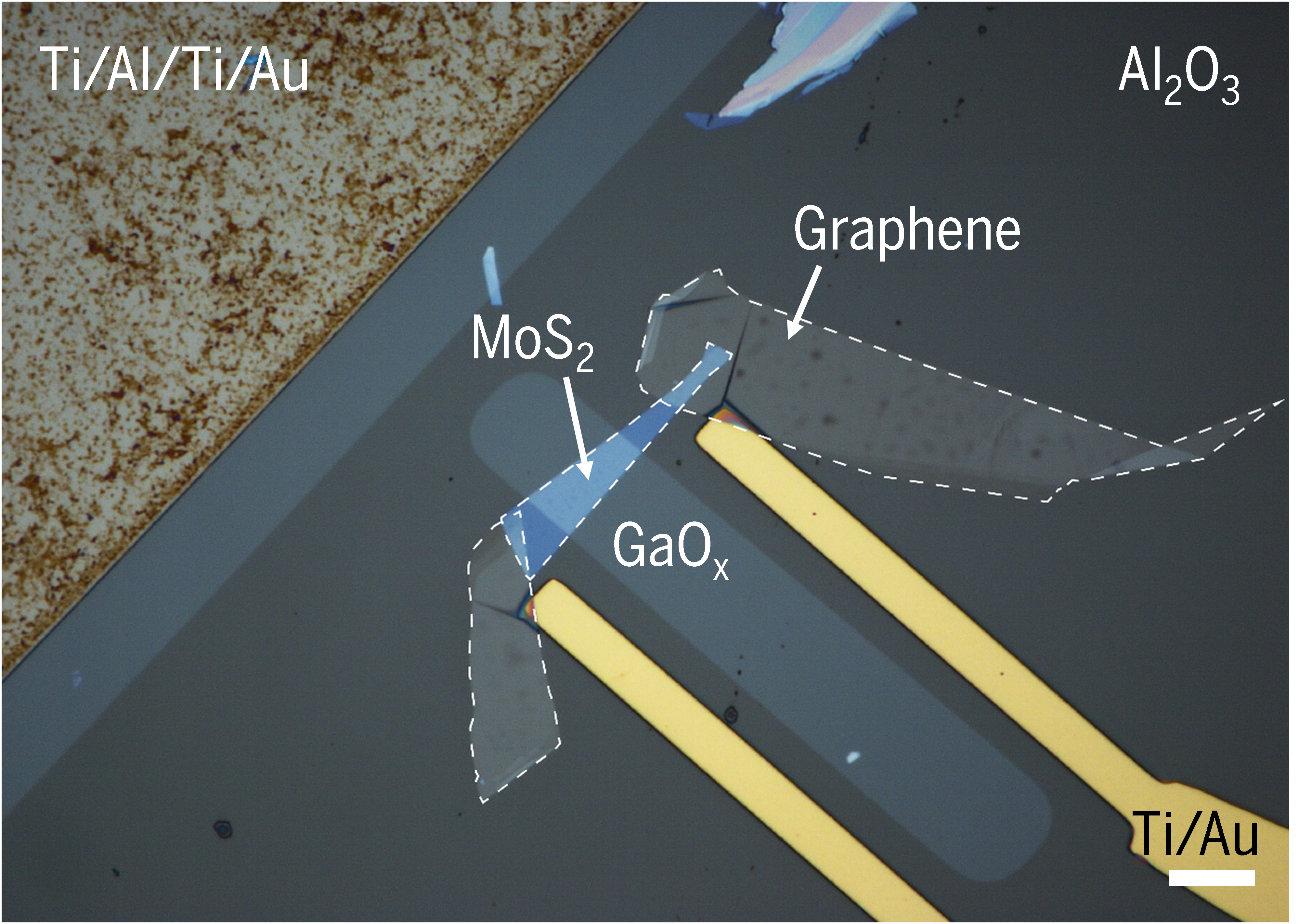
**

**Fig. S3.** **Optical microscope image of MoS_2_ transistor fabricated on a** **GaN/GaO_x_ heterostructure platform (Scale bar: 10 µm).**


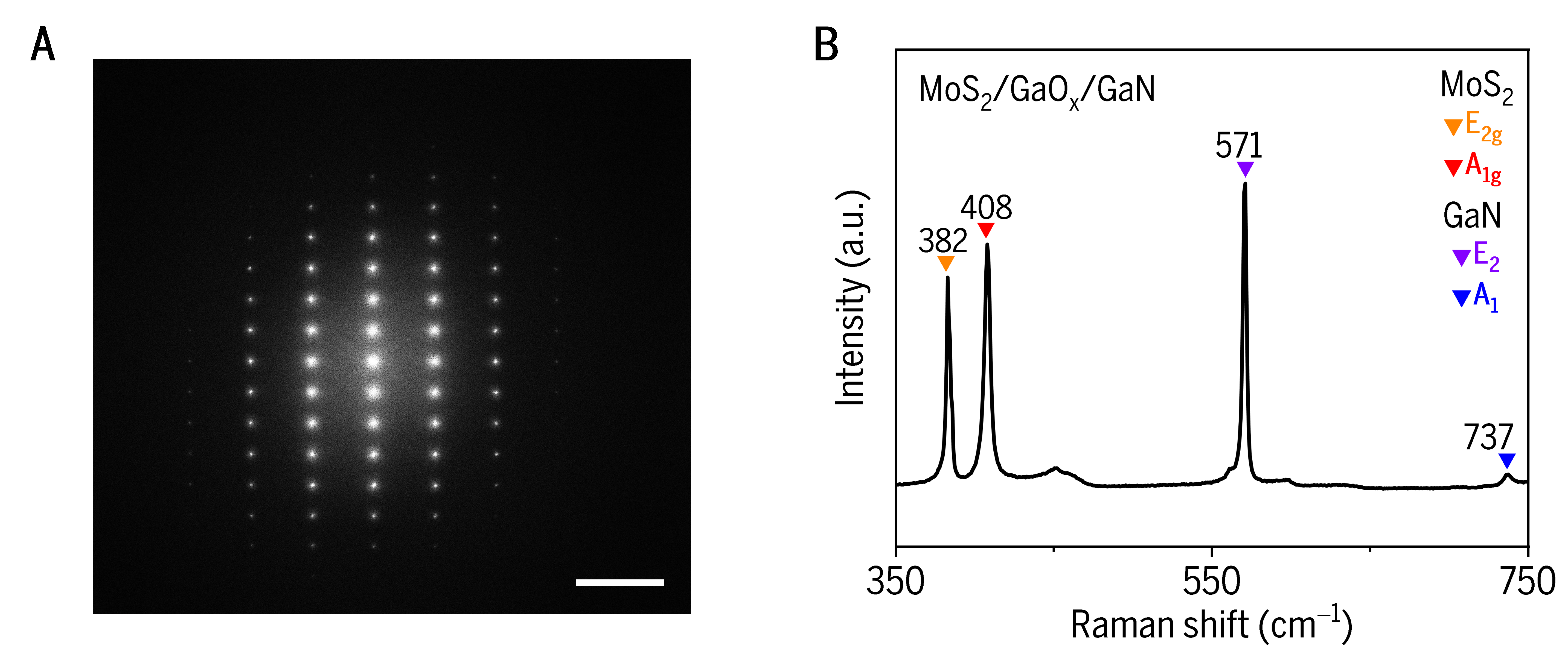


**Fig. S4.** **Characterization of MoS_2_/GaO_x_/GaN heterostructure.** (A) SAED pattern of GaN substrate (Scale bar: 5 nm^−1^). (B) Raman spectra.

**
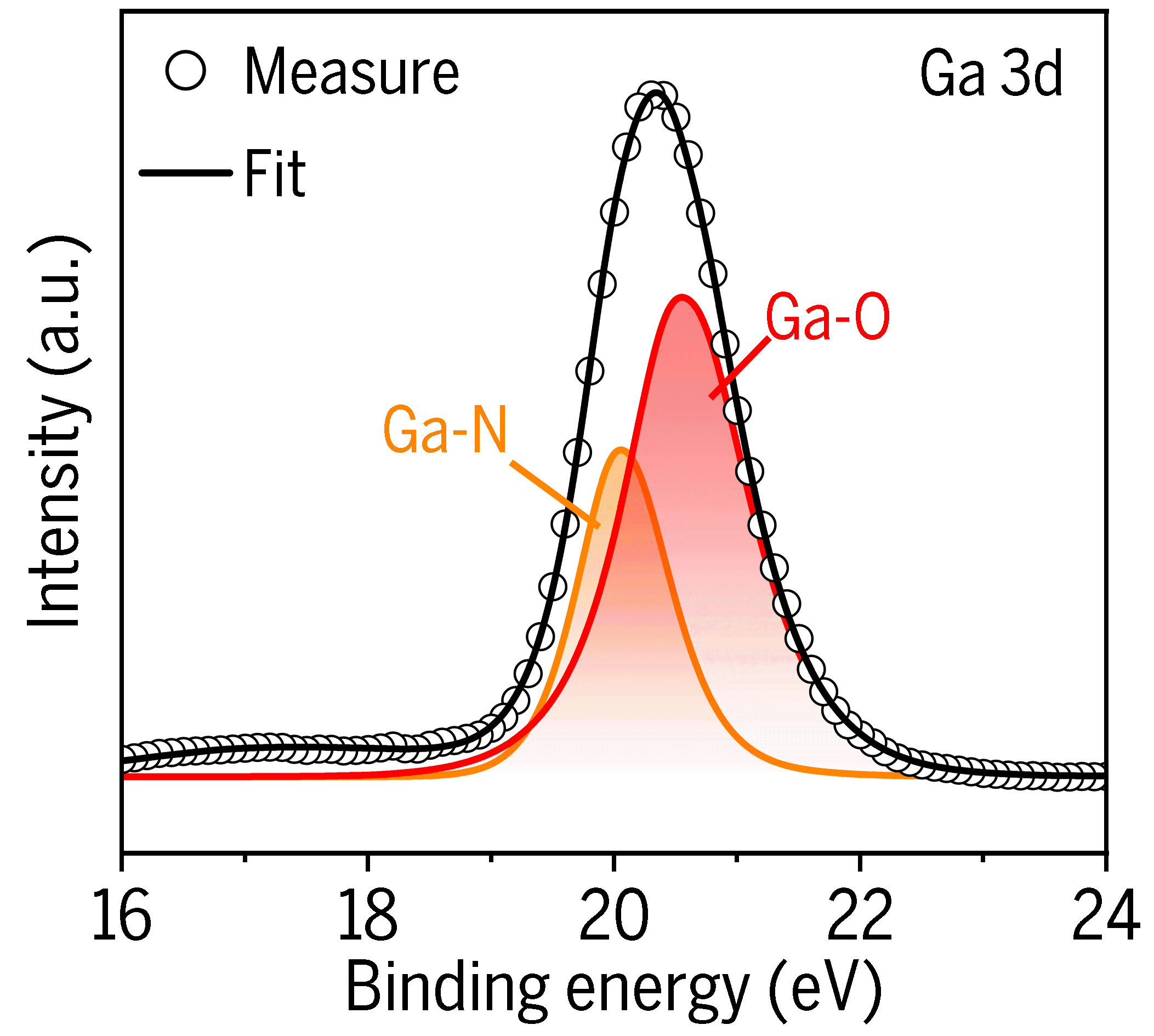
**

**Fig. S5.** **High-resolution XPS spectra of Ga 3d core-level for the GaO_x_ dielectric.** The experimental data can be well fitted by Ga-N (low-intensity) and Ga-O (high-intensity) bonding states.


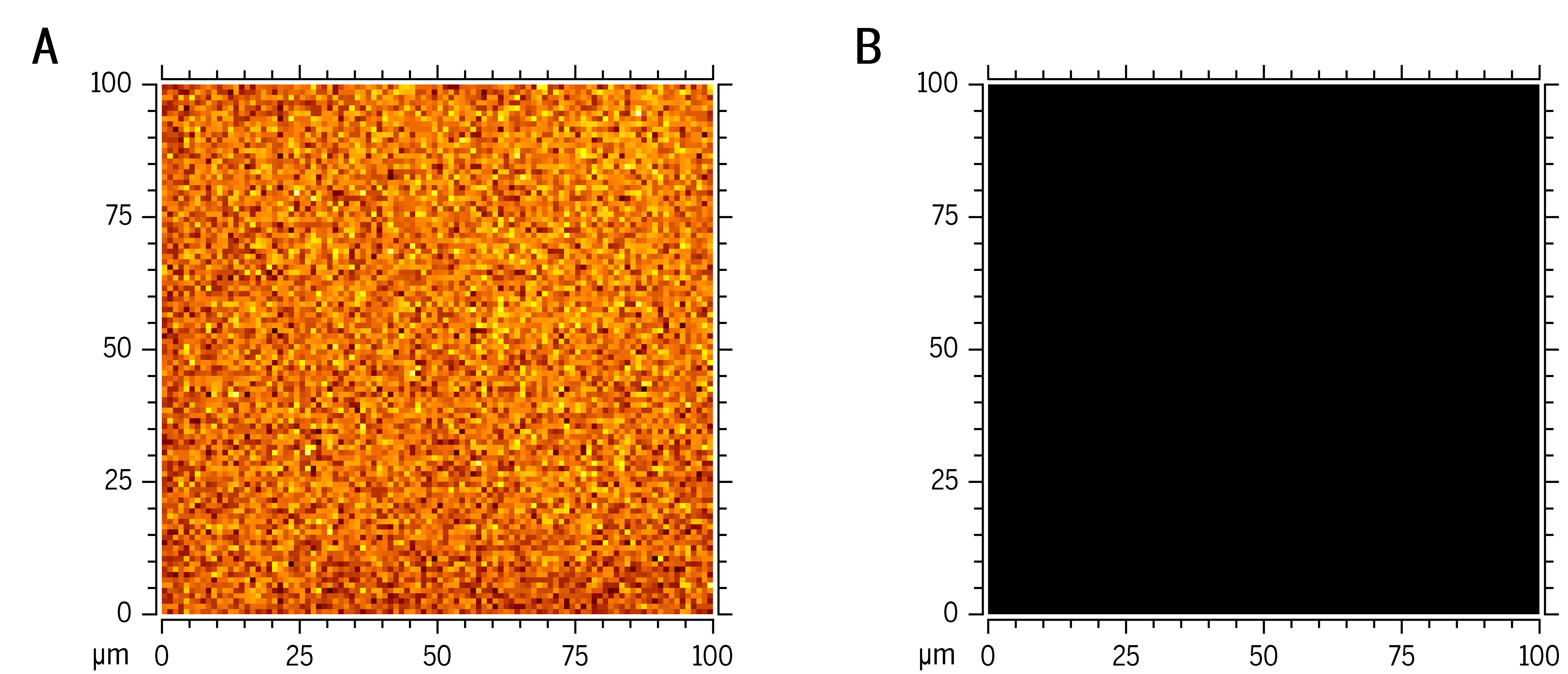


**Fig. S6.** **ToF-SIMS maps for the GaO_x_ dielectric.** (A) Ga-O content. (B) Ga-N content. The results demonstrate that the dielectric consists of pure Ga-O bonding, with negligible Ga-N content.


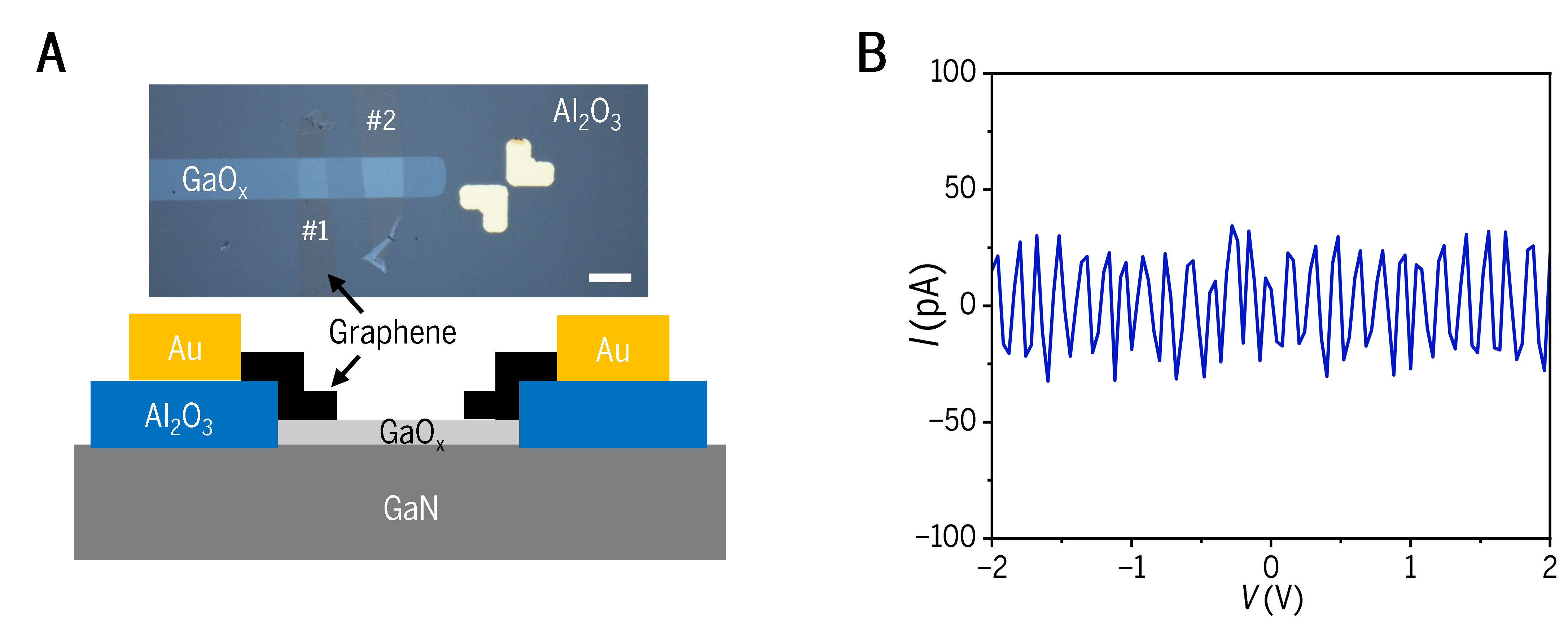


**Fig. S7. Electrical insulation performance of the GaO_x_ dielectric layer.** (A) Schematic and optical image of the electrode configuration for insulation test (Scale bar: 10 µm). (B) Current-voltage (*I-V*) characteristics of the GaO_x_ dielectric layer.

As shown in Fig. S7A, the test structure consists of two mechanically exfoliated graphene electrodes (#1 and #2) transferred onto the GaO_x_ dielectric surface. Electrode #1 (8.9 μm × 6.3 μm) served as the voltage application terminal, while electrode #2 (8.9 μm × 9.4 μm) was grounded, with an inter-electrode spacing of 9.2 μm. The current-voltage characteristics measured under a voltage sweep from −2 V to +2 V (Fig. S7B) demonstrate excellent insulation behavior of the GaO_x_ layer, confirming its reliable insulating performance within the 2 V operating range.


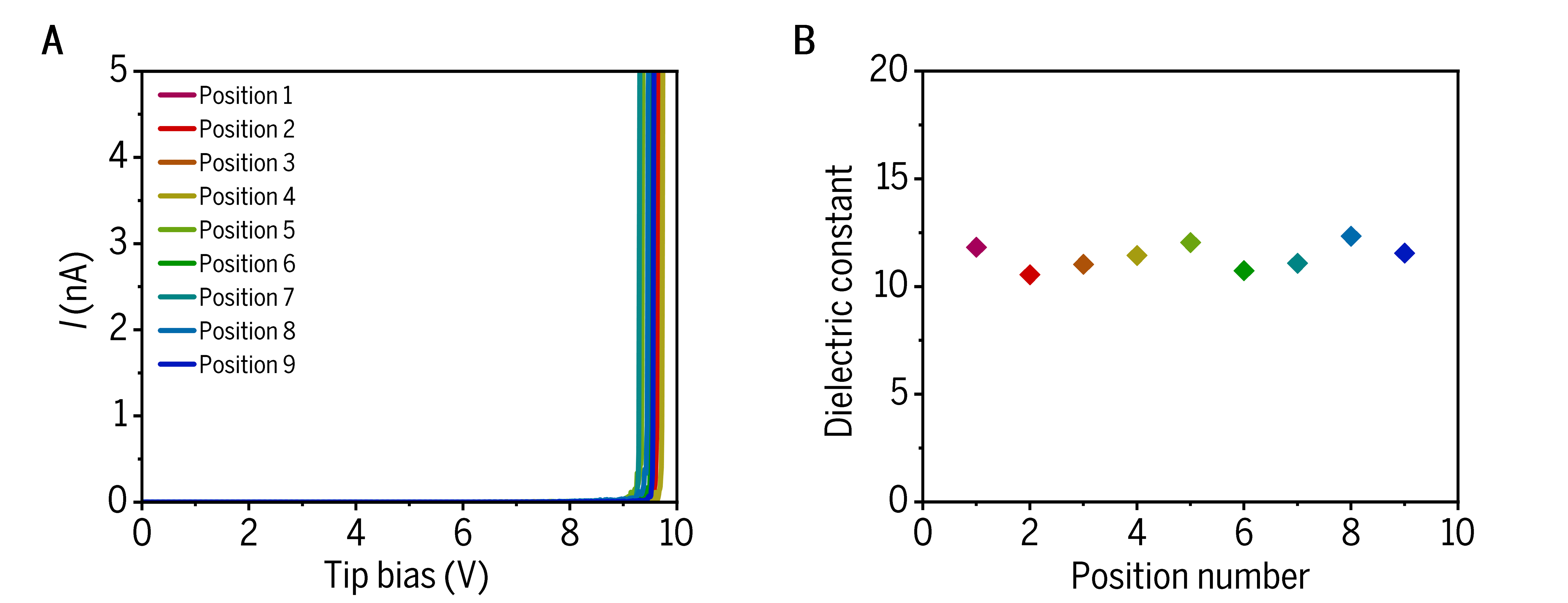


**Fig. S8. Determination of the dielectric constant of GaO_x_ dielectric.** (A) Current-voltage curves of GaO_x_ dielectric measured on the same sample (position 1, 2, 3, 4, 5) and different samples (position 6, 7, 8, 9). (B) Derived dielectric constant of corresponding samples in (A).

The *I-V* curve was obtained using AFM-based PF-TUNA characterization, and was fitted using the Schottky emission model. The fitting methods are as below.

The Schottky emission model for dielectric film can be described as:

$\text{J }\text{= }\frac{\text{4}\text{πq}{\text{k}_{\text{B}}}^{\text{2}}\text{m}^{\text{*}}}{\text{h}^{\text{3}}}\text{T}^{\text{2}}\text{e}^{\frac{\text{-q(}\text{φ}_{\text{B}}\text{-}\sqrt{\text{qE}/{\text{4}\text{π}\text{ε}_{\text{r}}\text{ε}_{\text{0}}}}\text{)}}{\text{k}_{\text{B}}\text{T}}}$ (1)

where *J* is the current density, *q* is the elementary charge of the electron, $\text{k}_{\text{B}}$ is the Boltzmann constant, $\text{m}^{\text{*}}$ is the effective electron mass in dielectric, ℎ is Planck’s constant, *T* is the temperature in Kelvin, $\text{φ}_{\text{B}}$ is the Schottky barrier height, *E* is the electric field, $\text{ε}_{\text{0}}$ is the permittivity of vacuum and $\text{ε}_{\text{r}}$ is the relative dielectric constant. The current density can be derived as follows:

$\text{J = }\frac{\text{I}}{\text{A}^{\text{*}}}$ (2)

where 𝐼 is the current and $\text{A}^{\text{*}}$ is the effective AFM tip contact area. We have rewritten Equation (1) as below:

$\text{I =}\text{ A}^{\text{*}}\frac{\text{4}\text{πq}{\text{k}_{\text{B}}}^{\text{2}}\text{m}^{\text{*}}}{\text{h}^{\text{3}}}\text{T}^{\text{2}}\text{e}^{\frac{\text{-q(}\text{φ}_{\text{B}}\text{-}\sqrt{\text{qE}/{\text{4}\text{π}\text{ε}_{\text{r}}\text{ε}_{\text{0}}}}\text{)}}{\text{k}_{\text{B}}\text{T}}}$ (3)

$\ln\text{(I)}\text{=}\ln\text{(α)}\text{+}\frac{\text{-q(}\text{φ}_{\text{B}}\text{-}\sqrt{\text{qE}/{\text{4}\text{π}\text{ε}_{\text{r}}\text{ε}_{\text{0}}}}\text{)}}{\text{k}_{\text{B}}\text{T}}$ (4)

To estimate the dielectric constant, we have further simplified the equation (4) as:

$\ln\text{(I)}\text{=}\frac{\text{q}\sqrt{\text{q}/{\text{4}\text{π}\text{ε}_{\text{r}}\text{ε}_{\text{0}}}}}{\text{k}_{\text{B}}\text{T}}\sqrt{\text{E}}\text{+ln} \text{(α)}\text{-}\frac{\text{q}\text{φ}_{\text{B}}}{\text{k}_{\text{B}}\text{T}}$ (5)

The electric field across the dielectric film is defined as:

$\text{E = }\frac{\text{V}}{\text{d}}$ (6)

where *V* is the applied voltage and *d* is the dielectric thickness. The thickness of GaO_x_ film was measured to be 3.2 nm. The linear fitting of $\ln\text{(I)}\text{ }$and $\sqrt{\text{E}}$ gives $\text{ε}_{\text{r}}$ of about 11.2 and R^2^= 0.925, indicating that our GaO_x_ is a high-κ dielectric.

In addition, 5 points were randomly selected on the same sample and 4 points were selected on different samples to test the *I-V* curves of GaO_x_ dielectric using the conductive AFM, and the results were similar, indicating that the GaO_x_ film had good uniformity.


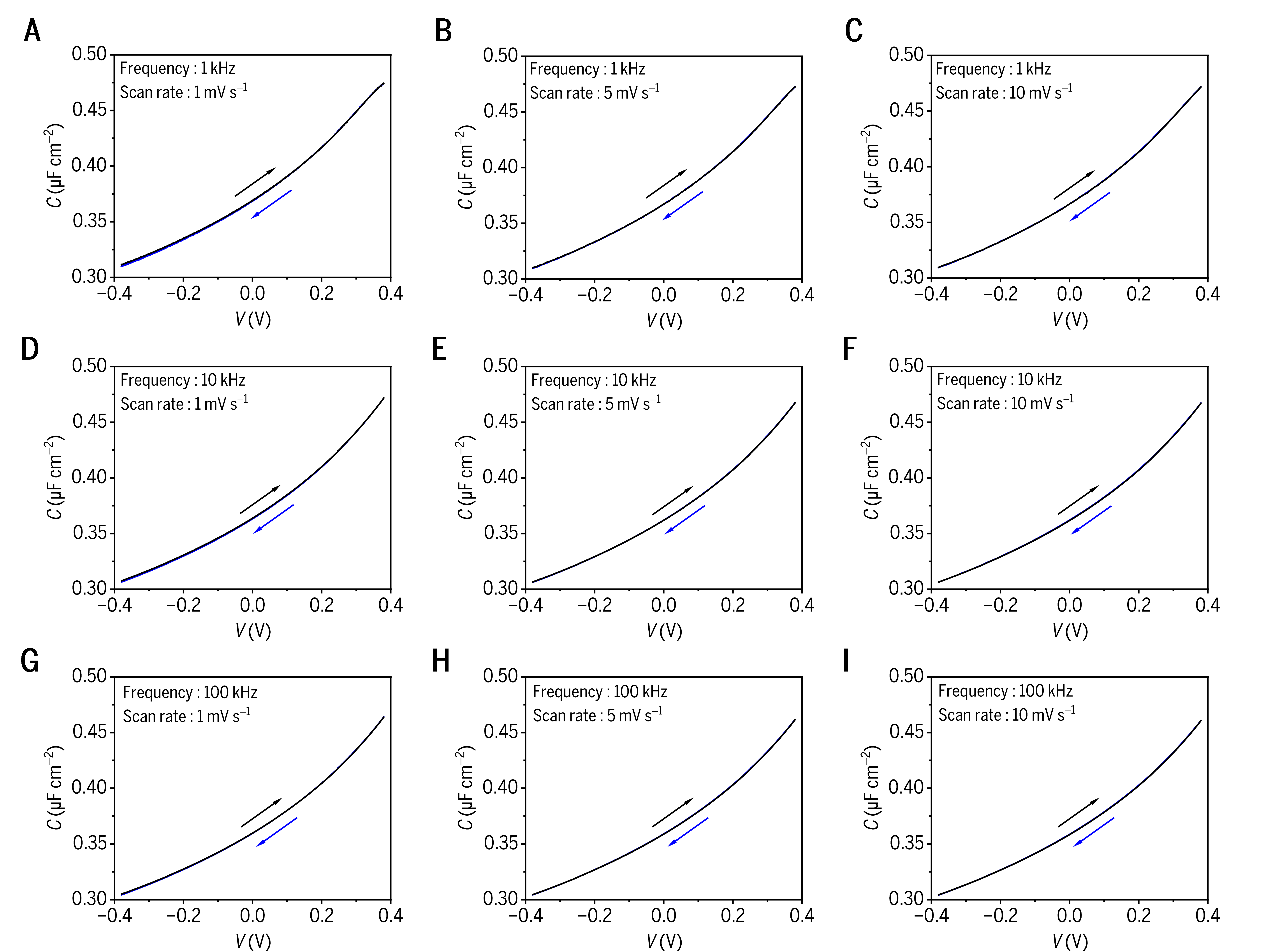


**Fig. S9.** ***C*-*V* characteristics of Au/GaO_x_/GaN structure at different frequencies and** **scanning rates.** (A-C) Scan rates of 1 mV s^−1^, 5 mV s^−1^ and 10 mV s^−1^, respectively, at a frequency of 1 kHz. (D-F) Scan rates of 1 mV s^−1^, 5 mV s^−1^ and 10 mV s^−1^, respectively, at a frequency of 10 kHz. (G-I) Scan rates of 1 mV s^−1^, 5 mV s^−1^ and 10 mV s^−1^, respectively, at a frequency of 100 kHz.


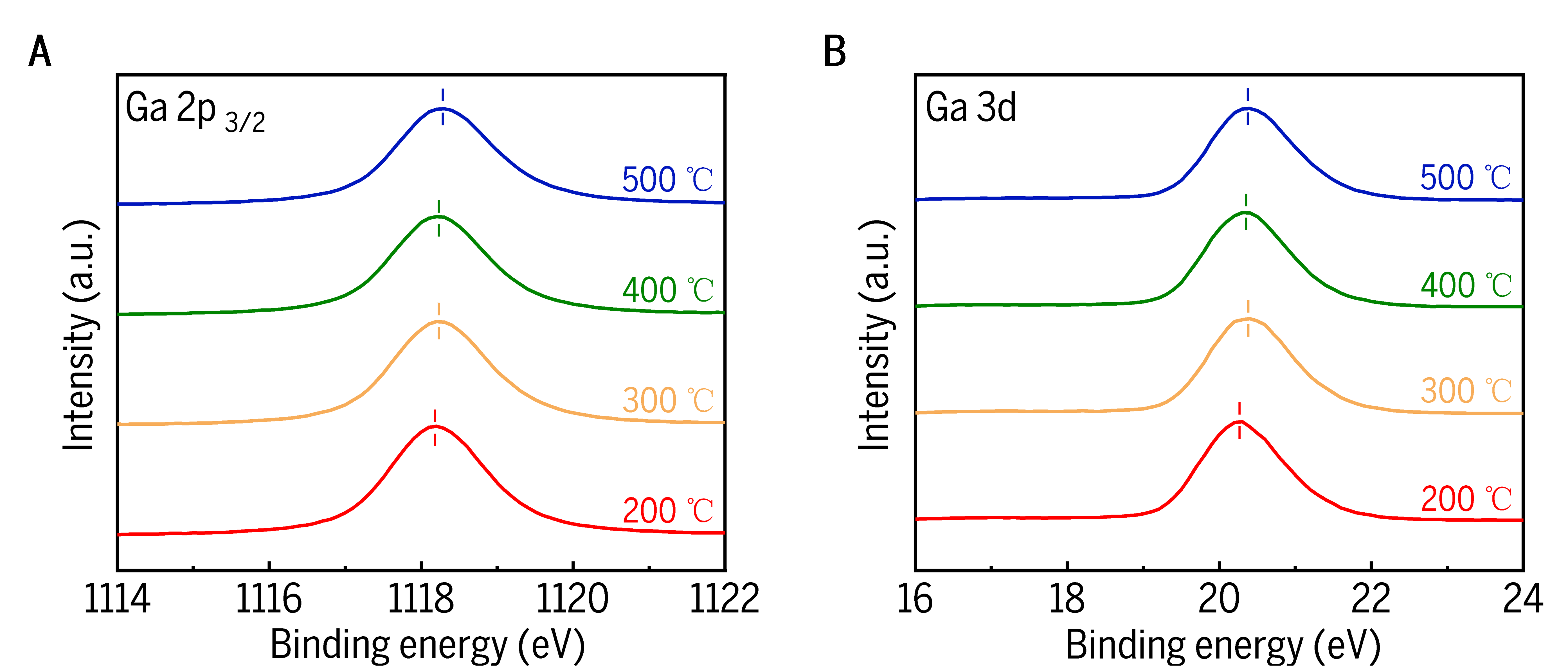


**Fig. S10. High-resolution XPS spectra of GaO_x_ dielectric fabricated at various oxidation temperatures for 60 min.** (A) Ga 2P _3/2_ core-level spectra. (B) Gap 3d core-level spectra. XPS analysis shows progressive shifts of the Ga 2p _3/2_ and Ga 3d peaks toward higher binding energies by 0.14 eV and 0.16 eV.

**
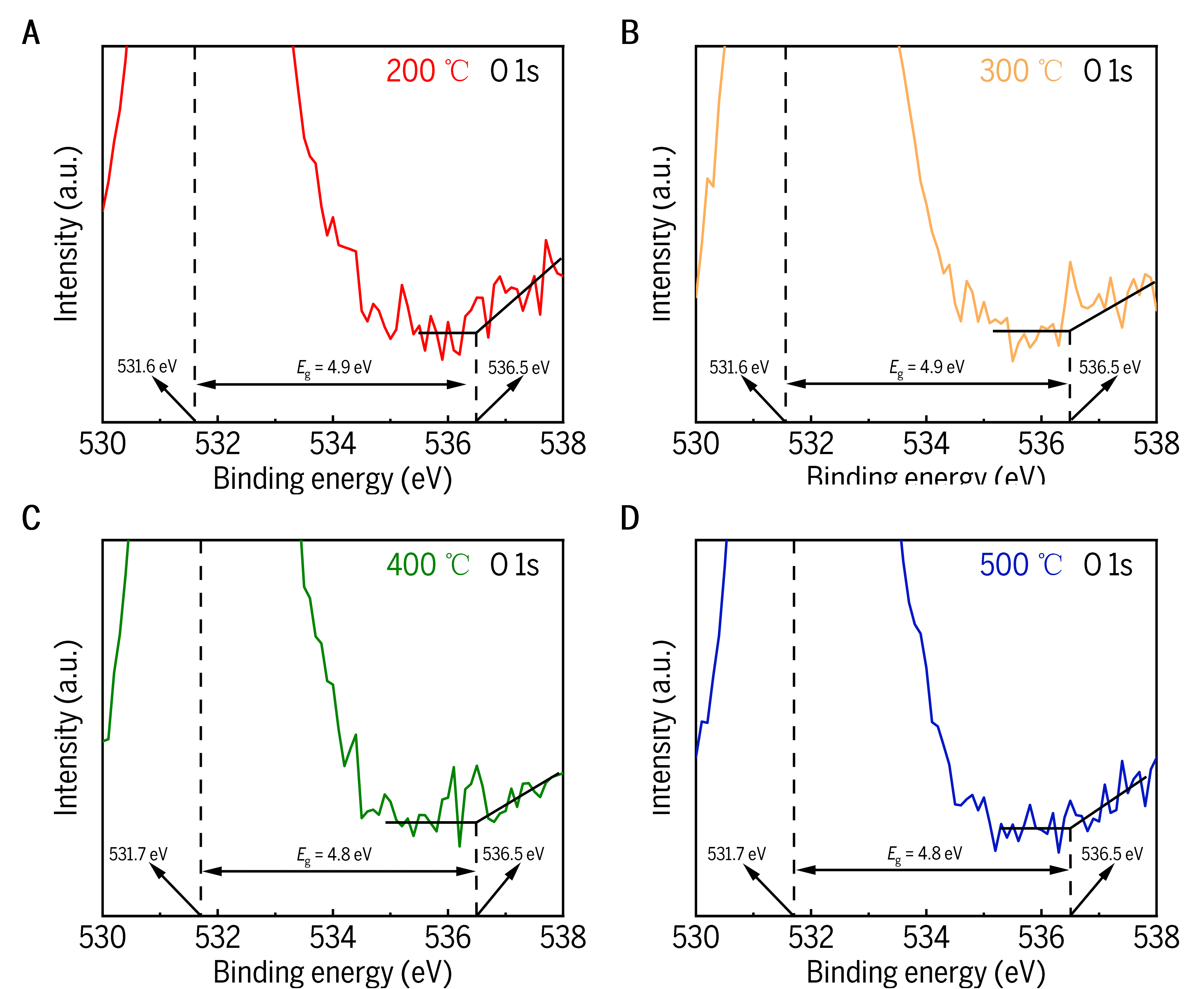
**

**Fig. S11.** **The band gap of GaO_x_ dielectric fabricated at various oxidation temperatures for 60 min was calculated through O 1s peak obtained by high resolution XPS.**


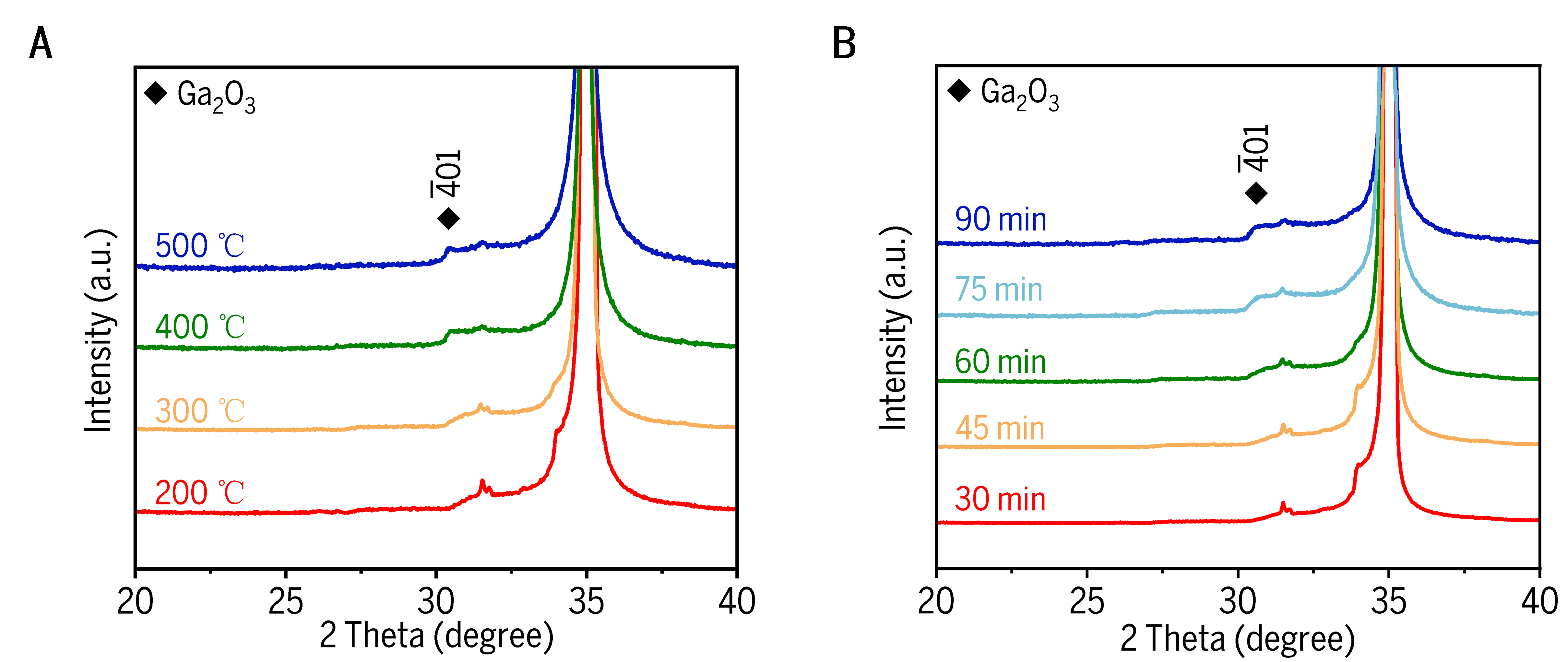


**Fig. S12.** **XRD patterns of GaO_x_ dielectric.** (A) XRD patterns of GaO_x_ dielectric fabricated at various oxidation temperatures for 60 min. (B) XRD patterns of GaO_x_ dielectric fabricated for various oxidation time at 300 ℃.


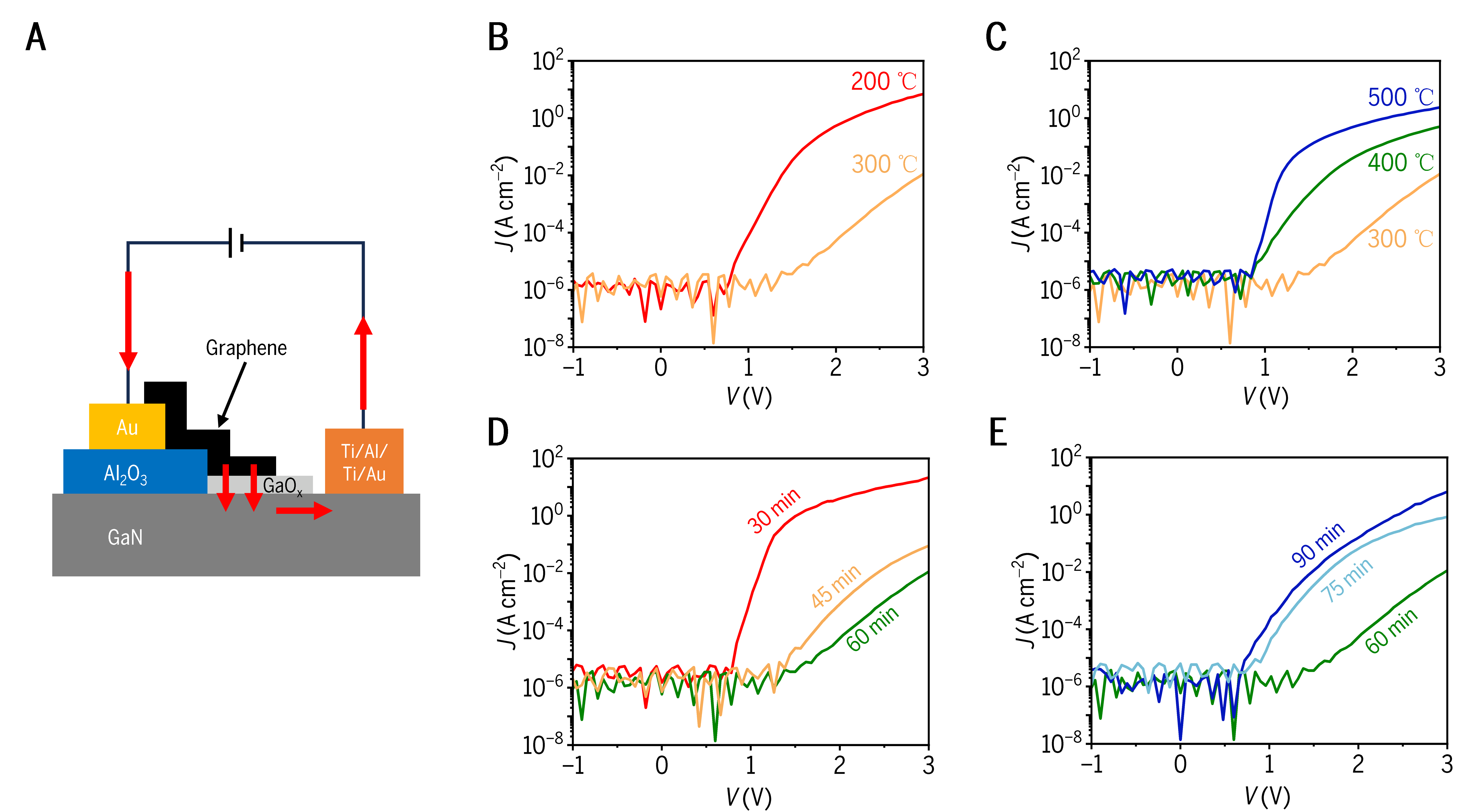


**Fig. S13.** **leakage current of GaO_x_ dielectric fabricated at different condition.** (A) Schematic diagram of current density measurement device. (B and C) Current density of GaO_x_ with different oxidation temperature at the same time (60 min). (D and E) Current density of GaO_x_ with different oxidation time at the same temperature (300 ℃).

Appropriate time and temperature were crucial for the fabrication of high-quality dielectric layers. Figure. S13A showed the schematic diagram of current density measurement device of GaO_x_. In Fig. S13B, the performance of GaO_x_ dielectric fabricated at 300 ℃ was better than 200 ℃, because a high-quality dielectric layer had not yet fully generated at 200 ℃ during this time. However, when the temperature further increased from 300 to 500 ℃, the performance decreased (Fig. S13C), because the transformation of crystal structure at high temperature (about 400 ℃). In Fig. S13D, the performance of the GaO_x_ dielectric fabricated at 60 min was better than 30 and 45 min, because a high-quality dielectric layer had not yet fully generated in a short period of time. With the time increased from 60 to 90 min, the performance decreased (Fig. S13E) because grains nucleated and grew abnormally below the transition temperature when in relatively high temperature for a long time.

**
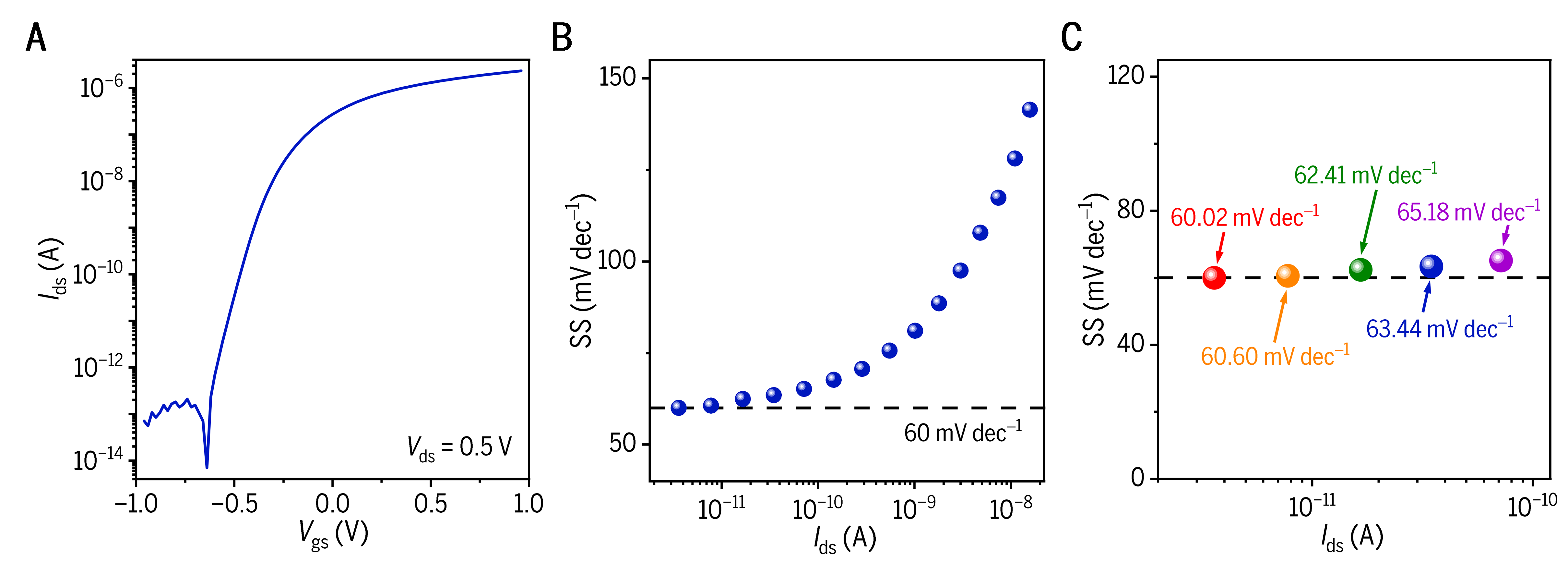
**

**Fig. S14. Analysis of the transfer characteristics and subthreshold swing (SS) of the MoS_2_ transistor.** (A) Transfer characteristic curve of the device. (B) The relationship between the subthreshold swing (SS) and the drain current (*I*_ds_), calculated from the data in panel (A). (C) A detailed view of the minimum SS value near the theoretical limit (60 mV dec^−1^), demonstrating its near-ideal gate control capability.


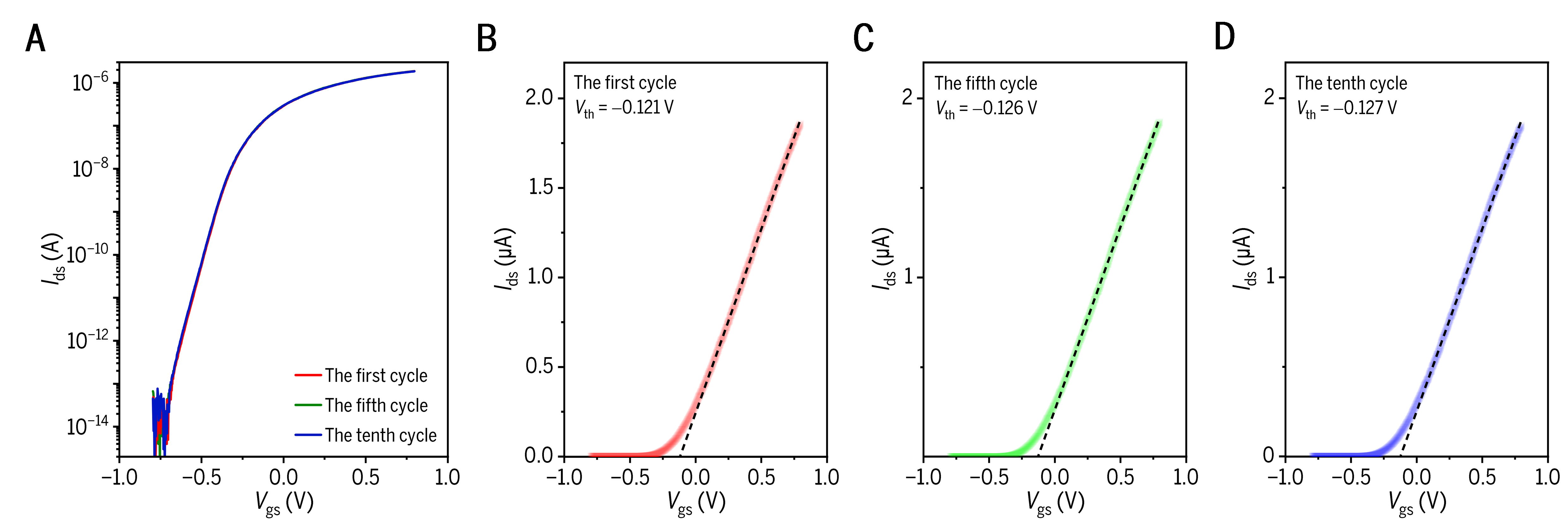


**Fig. S15.** **Threshold voltage stability over switching cycles****.** (A) Transfer characteristics at the first, fifth, and tenth cycles. (B-D) Corresponding threshold voltage extraction for the first, fifth, and tenth cycles, respectively.

We performed ten consecutive switching cycles to monitor the transfer characteristics. As shown in Fig. S15A, the overlayed curves from the first, fifth, and tenth cycles show nearly identical behavior. The corresponding threshold voltages, extracted in Fig. S15B-D, are −0.121 V, −0.126 V, and −0.127 V, respectively.


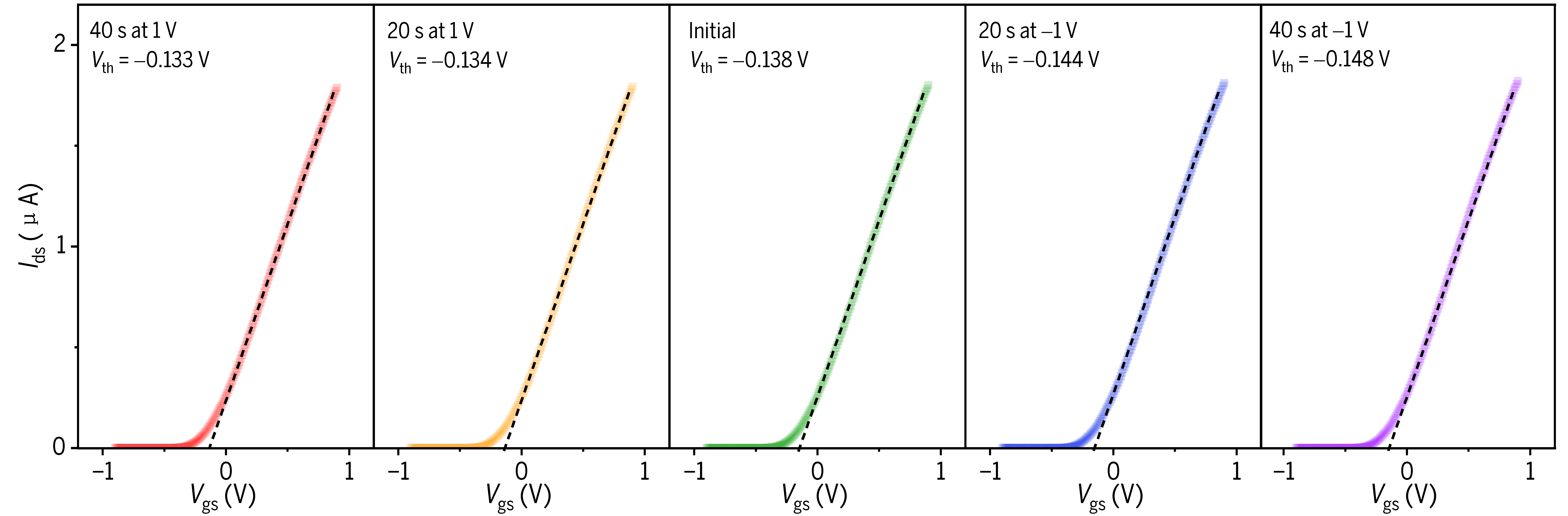


**Fig. S16**. **Transfer characteristics under various conditions for threshold voltage analysis.**

We have conducted systematic bias-stress stability tests on MoS_2_ transistors under gate voltages of ±1 V for stress durations of 20 s and 40 s, respectively. The results show that under positive bias stress (+1 V), the threshold voltage (*V*_th_) shifted positively from the initial value of −0.138 V, with shifts of +4 mV and +5 mV at 20 s and 40 s, respectively. Under negative bias stress (−1 V), the threshold voltage exhibited a continuous negative drift, with shifts of −5 mV and −10 mV at 20 s and 40 s, respectively.


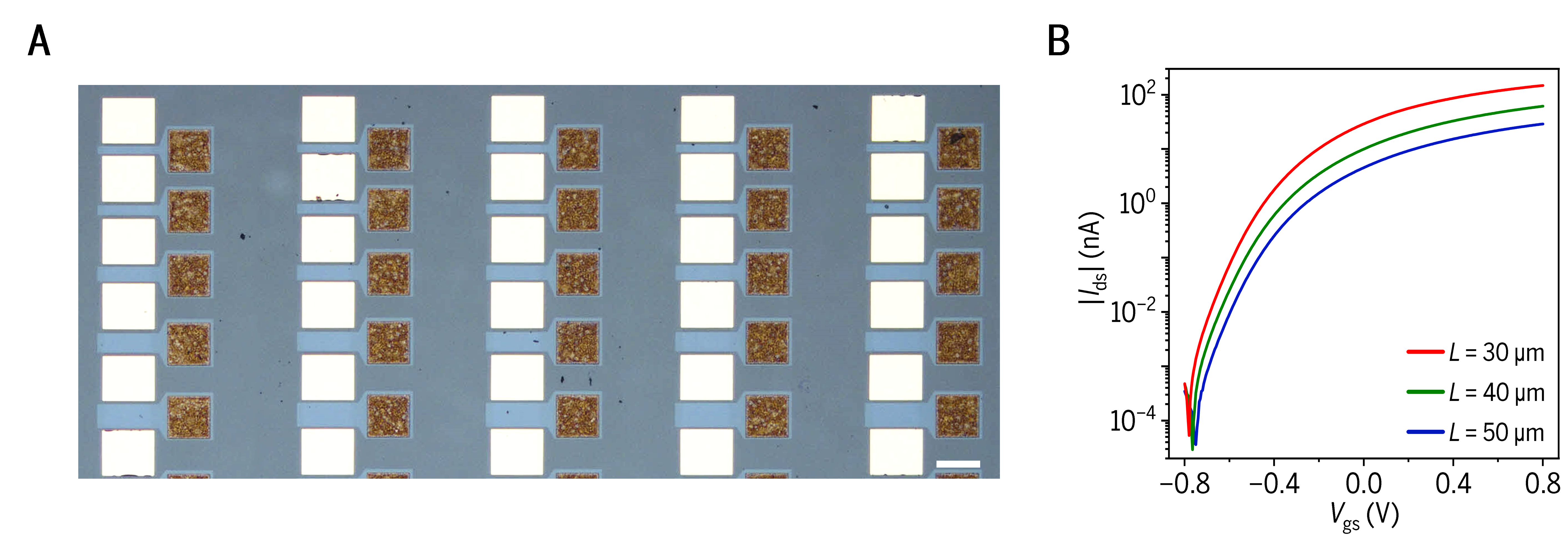


**Fig. S17. CVD MoS_2_ FET arrays fabricated on a GaN/GaO_x_ heterostructure platform.** (A) Optical image of FET arrays (Scale bar: 90 µm). (B) Transfer characteristic curves of transistors with different channel lengths.

**
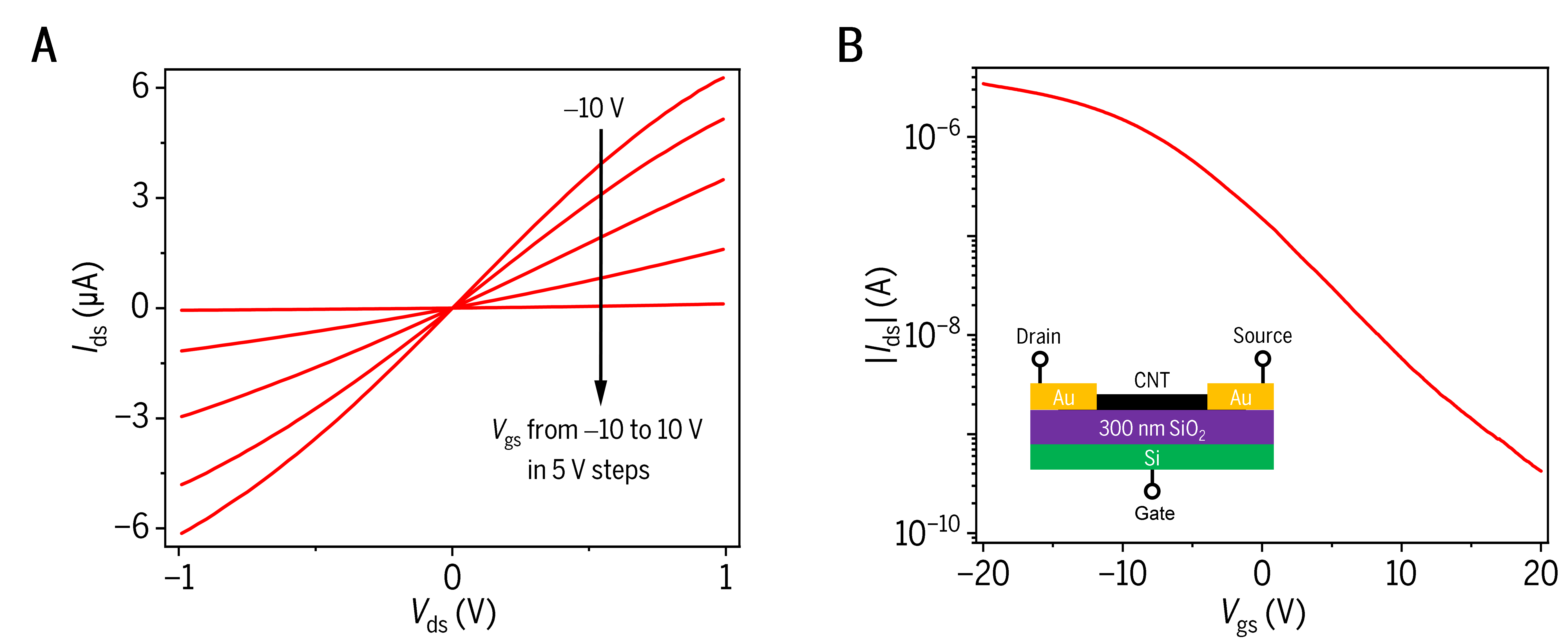
**

**Fig. S18. Electrical properties of CNT FET on the SiO_2_ substrate.** (A) Output characteristic curve of CNT transistor with *V*_gs_ increasing from −10 to 10 V at a step size of 5 V. (B) Transfer characteristic curve of the CNT transistor at *V*_ds_ = −1 V.

The channel length and width of CNT devices are 30 μm and 100 μm, respectively. CNT shows good contact with the gold electrodes at different gate voltages, and exhibits P-type characteristics.


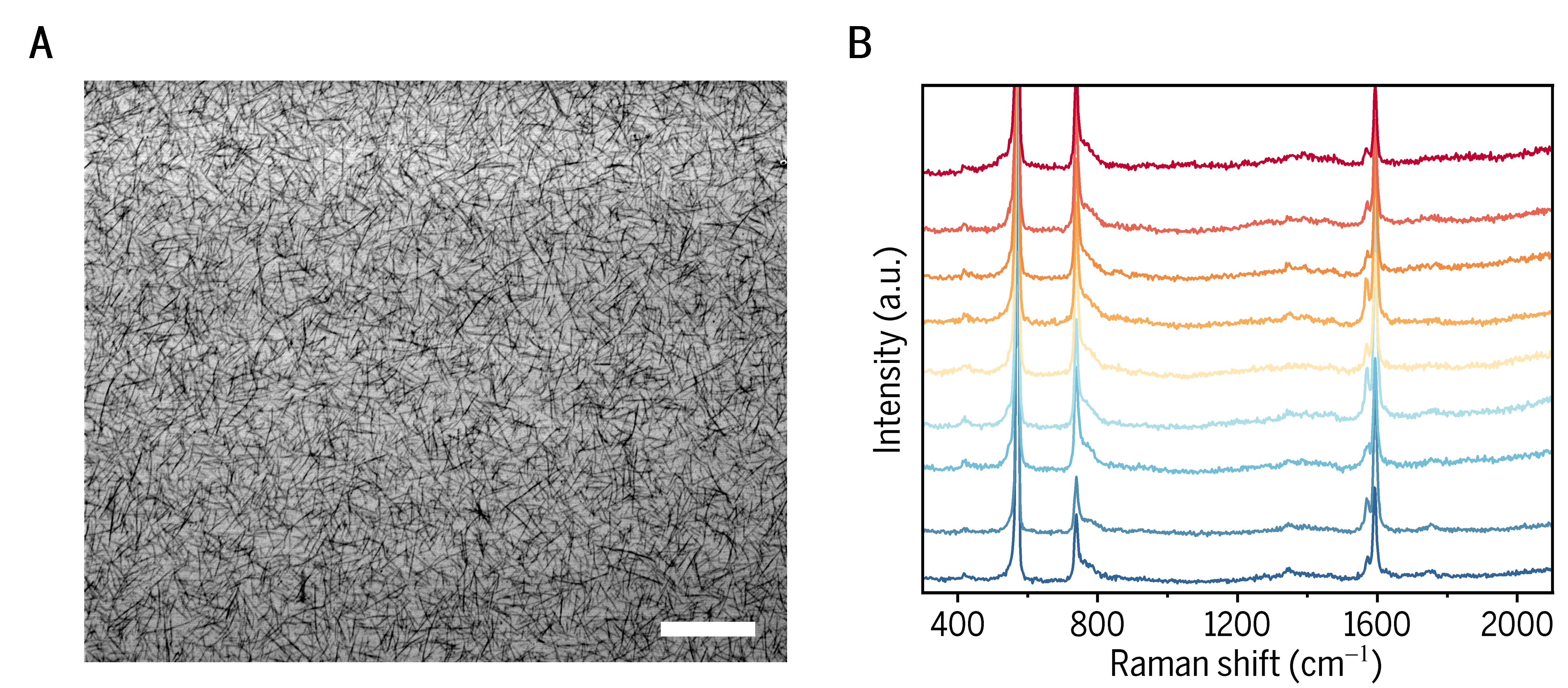


**Fig. S19. Characterization of the uniformity of the CNT film.** (A) SEM image showing the continuous and uniform network of the CNT film on the GaO_x_ dielectric (Scale bar: 5 µm). (B) Raman spectra collected from different locations on the CNT film.

SEM imaging reveals that the CNTs form a continuous, dense, and uniformly distributed interconnected network without any significant agglomeration or uncovered areas. Meanwhile, Raman spectroscopy demonstrates highly consistent intensity ratios of the characteristic peaks and minimal signal fluctuation across different locations.


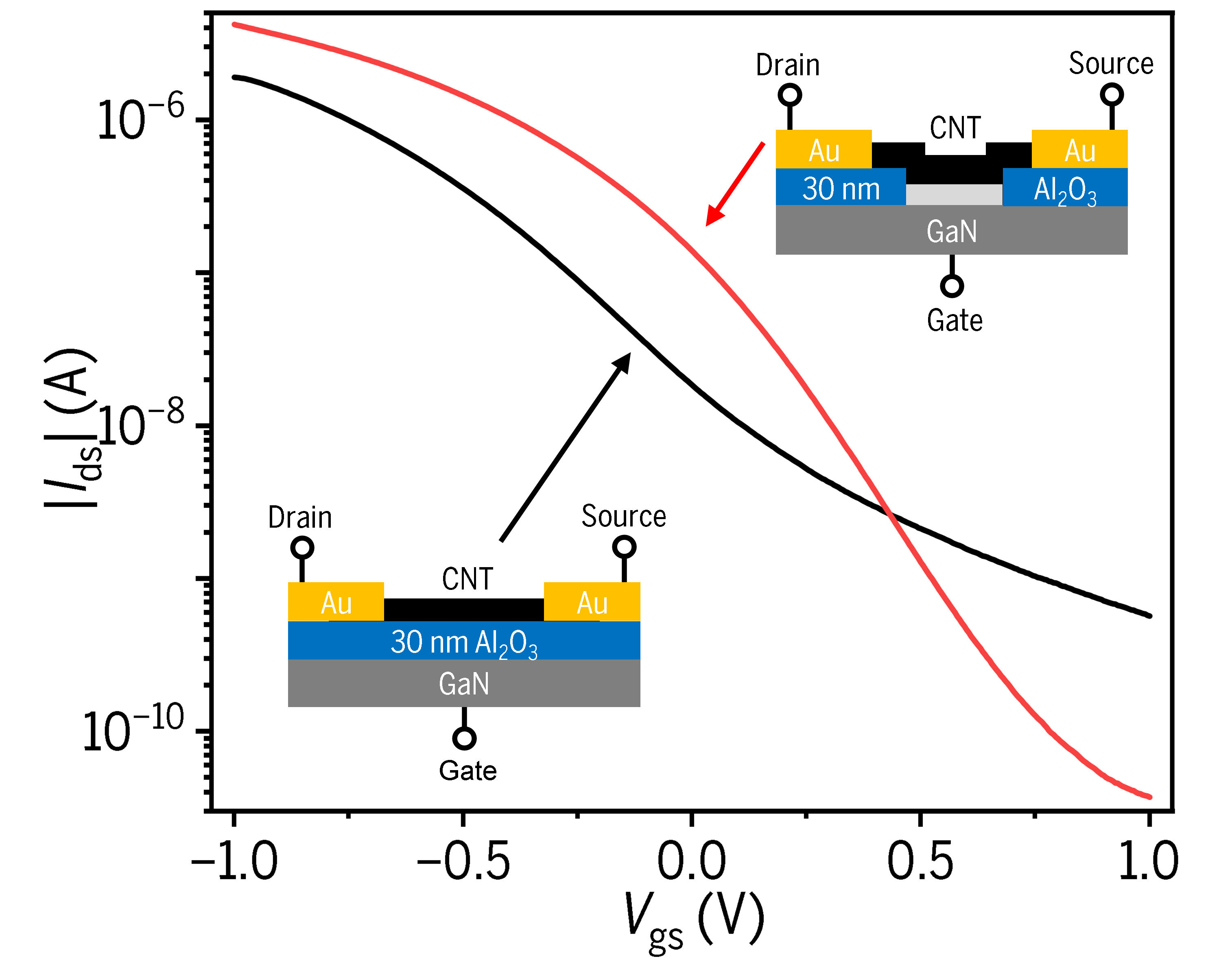


**Fig. S20.** **Transfer characteristic curves of CNT transistors with Al_2_O_3_ dielectric (black line) and GaO_x_ dielectric (red line) at *V*_ds_ = −1 V.**

The channel length and width of devices with different dielectric are 30 μm and 100 μm, respectively. The gate capacitance per unit area ($\text{C}_{\text{G}}$) is a critical factor in transistor gate control, primarily determined by the relative dielectric constant ($\text{ε}_{\text{r}}$) and the dielectric layer thickness ($\text{d}$). It is calculated by$\text{ }\text{C}_{\text{G}}\text{ = }\text{ε}_{\text{0}}\text{ε}_{\text{r}}\text{/d}$*.* GaO_x_ dielectric has an ultra-thin thickness of 3.2 nm and a high relative dielectric constant of 11.2, resulting in a significantly higher $\text{C}_{\text{G}}$ compared to Al_2_O_3_. Consequently, devices with GaO_x_ dielectric show higher gate control performance.


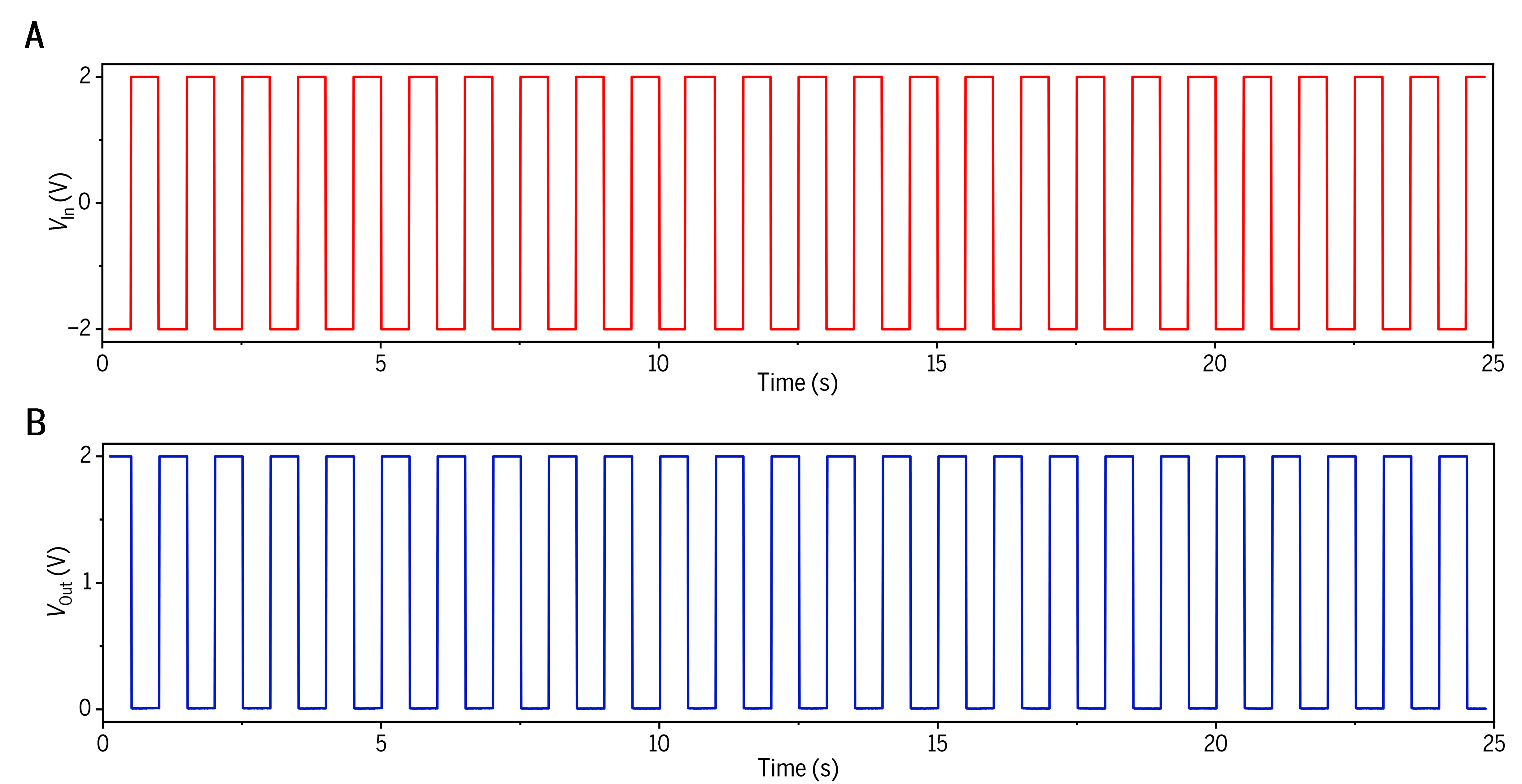


**Fig. S21**. **High operational stability of the MoS_2_/CNT complementary inverter.** (A) Input voltage signal applied to the inverter. (B) Output voltage response of the inverter.

During the test, a square-wave input voltage with a period of 1 second was applied (Fig. S21A), and the corresponding output voltage consistently exhibited a clear, stable, and phase-inverted square-wave response, as shown in Fig. S21B.


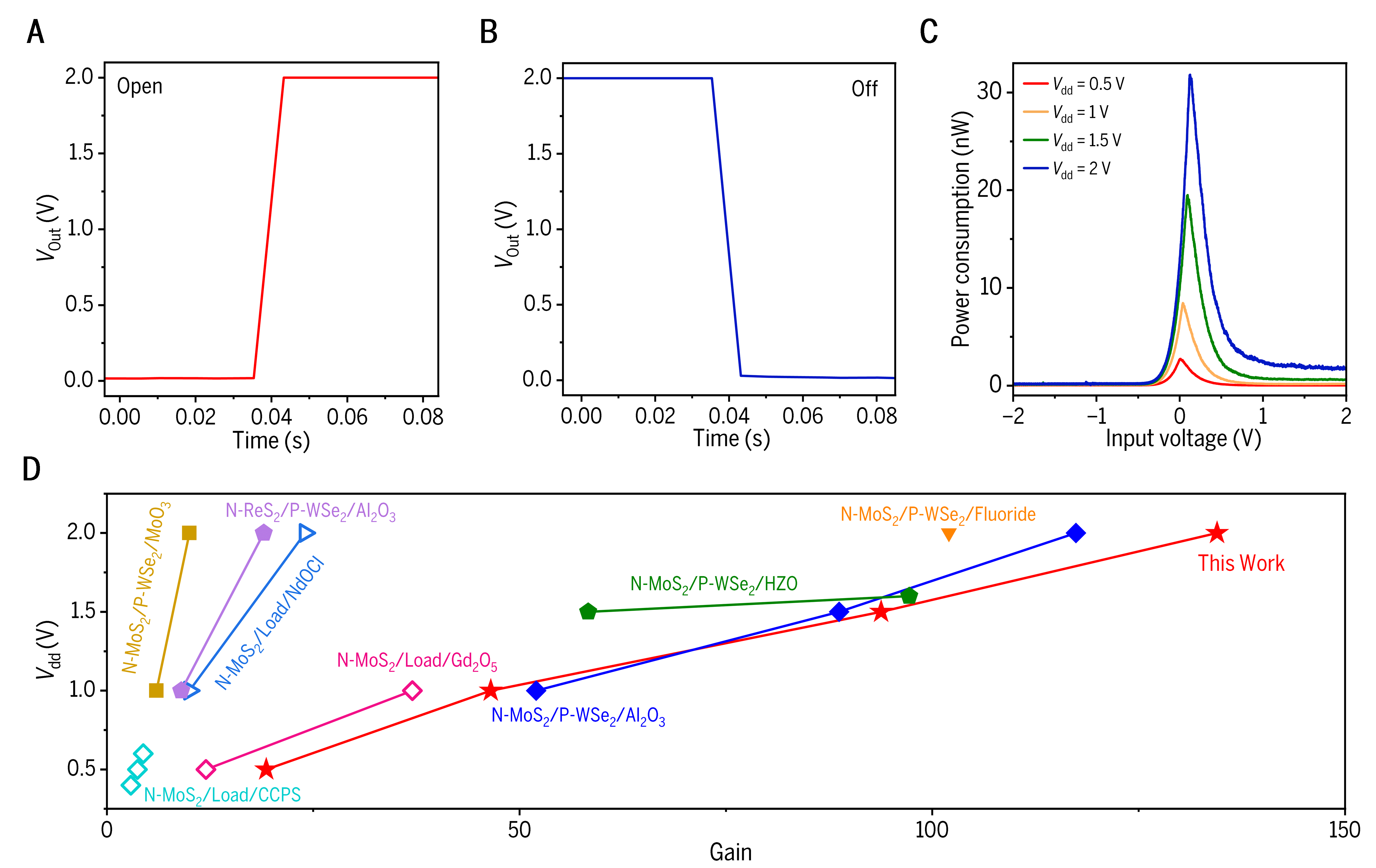


**Fig. S22. Performance characterization and benchmarking of the MoS_2_/CNT complementary inverter.** (A) Turn-on time. (B) Turn-off time. (C) Power consumption. (D) Performance benchmarking against other low-dimensional material-based inverters [1-8].

As shown in Fig. S22A and B, the inverter exhibits a rapid output voltage response upon input voltage switching, with measured rise and fall times of 6.3 ms and 6.2 ms, respectively. In terms of power consumption, power consumption under different supply voltages (*V*_dd_) demonstrate favorable low-power characteristics (Fig. S22C). Furthermore, a performance benchmark comparing the voltage gain of our inverter with other recently reported devices is summarized in Fig. S22D. The results demonstrate that our inverter achieves a competitive gain, highlighting the potential of the GaN/GaO_x_ heterostructure platform for advancing low-dimensional electronics.

**References**

1. Wu H, Xue JW, Wu Z, Liu JQ, Cheng YC, Xie ZD, Yan Z. Controllable p-type doping strategy for high-performance 2d material complementary inverters. *ACS Appl. Mater. Interfaces.* 2025;17(11): 17018-17025.
2. Xu WT, Huang J, Jiang JY, Liu P, Gong HX, Kang J, Jiang CB, Yang SX. Submillimeter-sized neodymium oxychloride single-crystal dielectrics for 2d electronics. *Adv. Mater.* 2025;e10240.
3. Yin L, Cheng RQ, Wan XH, Ding JH, Jia J, Wen Y, Liu XZ, Guo YZ, He J. High-κ monocrystalline dielectrics for low-power two-dimensional electronics. *Nat. Mater*. 2024;24(11): 197-204.
4. Fu T, Liu S, Niu BX, Shen WF, Hu CG, Peng RX, Liu K, Jiang CB, Yang SX. Low-power consumption anisotropic CMOS inverters based on n-ReS_2_ and p-WSe_2_. *Nano. Res.* 2025;18(3): 94907231.
5. Li XM, Xu SK, Zhang ZF, Yu ZQ, Pan ZD, Yang YJ, Lu XB, Huo NJ. Controllable growth of MoO_3_ dielectrics with sub-1 nm equivalent oxide thickness for 2d electronics. *Nat. Commun.* 2025;16: 6758.
6. Chen H, Long YF, Zhang SY, Liu K, Chen MF, Zhao JX, Si MW, Wang L. Van der waals ferroelectric CuCrP_2_S_6_-enabled hysteresis-free negative capacitance field-effect transistors. *Adv. Mater.* 2025;37(18): 2419125.
7. Meng K, Li ZY, Chen P, Ma XY, Huang JW, Li JY, Qin F, Qiu CY, Zhang YL, Zhang D, et al. Superionic fluoride gate dielectrics with low diffusion barrier for two-dimensional electronics. *Nat. Nanotechnol.* 2024;19(5): 932-940.
8. Lin CY, Chen BC, Liu YC, Kuo SF, Tsai HC, Chang YM, Kuo CY, Chang CF, Chen JH, Chu YH, et al. Integration of freestanding hafnium zirconium oxide membranes into two-dimensional transistors as a high-κ ferroelectric dielectric. *Nat. Electron.* 2025;8(6): 560-570.
